# 40 Years of Research on Polybrominated Diphenyl Ethers (PBDEs)—A Historical Overview and Newest Data of a Promising Anticancer Drug

**DOI:** 10.3390/molecules26040995

**Published:** 2021-02-13

**Authors:** Laura Schmitt, Ilka Hinxlage, Pablo A. Cea, Holger Gohlke, Sebastian Wesselborg

**Affiliations:** 1 Institute for Molecular Medicine I, Medical Faculty and University Hospital Düsseldorf, Heinrich Heine University Düsseldorf, Universitätsstraße 1, 40225 Düsseldorf, Germany; laura.schmitt@med.uni-duesseldorf.de (L.S.); ilka.hinxlage@hhu.de (I.H.); 2 Institute for Pharmaceutical and Medicinal Chemistry, Heinrich Heine University Düsseldorf, Universitätsstraße 1, 40225 Düsseldorf, Germany; Pablo.cea.Medina@hhu.de (P.A.C.); Gohlke@uni-duesseldorf.de (H.G.); 3 John von Neumann Institute for Computing (NIC), Jülich Supercomputing Centre (JSC) & Institute of Biological Information Processing—Structural Biochemistry (IBI-7), Forschungszentrum Jülich GmbH, Wilhelm-Johnen-Str., 52425 Jülich, Germany

**Keywords:** PBDE, *Dysidea* sp., anticancer, apoptosis, intrinsic mitochondrial pathway, P01F08

## Abstract

Polybrominated diphenyl ethers (PBDEs) are a group of molecules with an ambiguous background in literature. PBDEs were first isolated from marine sponges of *Dysidea* species in 1981 and have been under continuous research to the present day. This article summarizes the two research aspects, (i) the marine compound chemistry research dealing with naturally produced PBDEs and (ii) the environmental toxicology research dealing with synthetically-produced brominated flame-retardant PBDEs. The different bioactivity patterns are set in relation to the structural similarities and dissimilarities between both groups. In addition, this article gives a first structure–activity relationship analysis comparing both groups of PBDEs. Moreover, we provide novel data of a promising anticancer therapeutic PBDE (i.e., 4,5,6-tribromo-2-(2′,4′-dibromophenoxy)phenol; termed P01F08). It has been known since 1995 that P01F08 exhibits anticancer activity, but the detailed mechanism remains poorly understood. Only recently, Mayer and colleagues identified a therapeutic window for P01F08, specifically targeting primary malignant cells in a low µM range. To elucidate the mechanistic pathway of cell death induction, we verified and compared its cytotoxicity and apoptosis induction capacity in Ramos and Jurkat lymphoma cells. Moreover, using Jurkat cells overexpressing antiapoptotic Bcl-2, we were able to show that P01F08 induces apoptosis mainly through the intrinsic mitochondrial pathway.

## 1. Introduction

The search for new bioactive substances, which can overcome intrinsic or acquired resistance, are core topics of pharmaceutical research. Thus, there is a constant need for new types of resistance-breaking drugs due to the spread of multidrug-resistant microorganisms and tumors. Ecological niches under high evolutionary pressure often yield bioactive compounds with high antibacterial or antineoplastic capacity (e.g., coral reefs). These compounds and their analogs from stress-exposed marine organisms or fungal endophytes could serve as a pool for new, potentially active compounds to elucidate the modes of action and overcome resistance at the molecular level. The global pharmaceutical market amounts to 1.1 trillion US dollars [1]. About 65 percent of all 1,211 small-molecule drugs approved by the FDA between 1981 and 2014 are based on natural products, including derivatives and synthetic drugs with pharmacophores or mimics of natural products [2]. Natural products offer a high degree of structural diversity, including highly complex carbon scaffolds, along with advantageous pharmacokinetic and pharmacodynamic properties compared to synthetic substances due to their formation and evolution in biological systems [3].

Marine organisms are an especially diverse and rich source of natural products, with manifold bioactivities ranging from the inhibition of growth to the induction of apoptosis [4,5]. A huge step for the production of natural compounds during evolution in marine organisms was the acquisition of symbiotic bacteria. These serve as factories for the synthesis of unique bioactive compounds [6]. Especially the symbiosis of sponges with bacteria serves as a treasure chest for the acquisition of novel natural compounds. In this context, Peter Proksch has dedicated a better part of his research career to the isolation and characterization of natural product drugs from marine organisms [7,8,9,10]. Approximately 40–60% of the total sponge mass consists of bacteria [11,12], and the high density of symbiotic microbes in sponge tissues is considered the main source of many secondary metabolites found in sponge extracts [13,14]. However, the relationship between bacteria and sponges in terms of symbiotic benefits remains poorly understood [6]. Sponges are thought to be the oldest, simplest multicellular animals possessing many cell types of various functions. However, they lack a true tissue and comprise a kind of mesenchyme—the so called mesohyl—that mainly consists of collagen. In general, sponges can be classified based on their skeletal components or the lack thereof, which can be made up of either separate or fused spicules of calcium carbonate or silicon dioxide, or collagen fibers and filaments (building an organic skeleton). The symbiotic relationship between sponges and bacteria is possible because sponges contain lectins (sugar-binding proteins important in cellular recognition). These lectins allow bacteria to coexist on and in sponge tissue due to the lectin-containing binding site for some symbionts [6,15,16].

This article aims to summarize data about the bioavailability and diversity of polybrominated diphenyl ethers (PBDEs), a class of marine natural products, mainly extracted from marine sponges. Additionally, the newest data of a unique PBDE named P01F08 (**1**), which showed promising antineoplastic capacity, will be presented, followed by a structure activity relationship (SAR) analysis.

The PBDE P01F08 (4,5,6-tribromo-2-(2′,4′-dibromophenoxy) phenol) (**1**) [all molecules (**1**)–(**44**) in this publication are listed in Supplementary Table S1 and corresponding structures are shown in Supplementary Figure S1] was identified in a previously reported screening of 300 natural compounds from the biobank of Peter Proksch at the Institute of Pharmaceutical Biology and Biotechnology at the Heinrich Heine University Duesseldorf [17] and had been isolated from the marine sponge *Dysidea* species (sp.) [18]. The compound showed extensive antineoplastic activity on T cell leukemia (Jurkat J 16) and B cell lymphoma (Ramos) cell lines [17]. Moreover, it was postulated to be of therapeutic relevance for patients with acute myeloid leukemia (AML) as it affected primary malignant cells 3.2-fold stronger than their healthy counterparts [17].

## 2. Nomenclature of Polybrominated Diphenyl Ethers

PBDEs are unique molecules containing diphenyl ether scaffolds and hydroxy (-OH) or methoxy (-MeO), ether, and bromine (-Br) functional groups [19]. They can be separated in hydroxylated brominated diphenyl ether derivatives (OH-PBDEs/OH-BDEs), their methoxylated counterparts (MeO-PBDEs/MeO-BDEs), and di-hydroxylated derivatives (dioxins or di-OH-BDEs). In general, PBDEs were thought to be derived from a chemical transformation of anthropogenically produced polybrominated flame retardant chemicals of similar structure [20,21,22]. The origin of MeO-PBDEs has been unclear for a long time. They have been found bioaccumulated in the tissues of a variety of aquatic organisms but it was proven via ^14^C-measurement that the most frequently observed PBDE derivatives in animal tissue (e.g., 6-MeO-BDE-47 (**2**) and 2-MeO-BDE-68 (**3**), which are also widely detected in marine metabolomes) are of natural origin [20,21].

As mentioned in the introduction, OH-PBDEs and MeO-PBDEs are abundant across all trophic stages of marine life, from plants [23], algae [24,25,26] and invertebrates [27,28,29,30], to marine mammals [20,31,32]. In the following, we will focus on PBDEs isolated from sponges.

## 3. Biodiversity of PBDEs

At first, sponges that are a rich source of PBDEs had to be classified. They belong to the kingdom *Animalia*, phylum *Porifera*, class *Demospongiae* (meaning they are ′horny′ sponges, having a skeleton of organic fibers containing spongin, which is a collagen-like material [33]), the order of *Dictyoceratida*, and the family *Dysideidae* (for detailed information about the first describers refer to the World Register of Marine Species).

The marine sponges of the *Dysideidae* family are distributed in tropical and subtropical waters around the world [34]. They have been proven to be prolific producers of a variety of secondary metabolites such as bromophenols, sesquiterpenes, sesterpenes, sterols, poly-chlorinated compounds, and diphenyl ethers (listed in [35] and [36]). Several sponge species found to produce PBDEs and their isolation locations are summarized in Table 1.

It was assumed in several sources that not the sponges themselves are the producers of polyhalogenated (either polybrominated or polychlorinated) compounds [15,27,42,52,53].

Selected sponge-derived metabolites and their putative production source are exemplarily listed in Flatt et al. [52]. The authors grouped brominated phenols mainly found in *Dysidea herbacea* with the cellular source of filamentous cyanobacteria [27]. It has been known since 1993 that *Dysidea herbacea* harbor *Oscillatoria spongeliae* cyanobacteria [27,53]; cell localization experiments demonstrated that they are likely producers of polychlorinated peptides. Flatt et al. clearly localized the expression of biosynthetic genes required for the production of polychlorinated peptides in *Dysidea herbacea* by deposition fluorescence in situ hybridization (CARD-FISH) [52]. The symbiotic cyanobacteria *Oscillatoria spongeliae* comprise around 30–40% of the total sponge biomass [52].

Two bacterial specimens of the *Vibrio* genus were found to harbor the same polybrominated diphenyl ethers as those obtained from organic extracts of the *Dysidea* species in 1991, although the symbiont was not demonstrated to produce the compounds [42]. For *Dysidea herbacea*, a polybrominated diphenyl ether was found to be deposited as conspicuous crystals throughout the sponge tissue. A dominant prokaryotic endosymbiont was found in the mesohyl of the sponge and identified as *Oscillatoria spongeliae*, a vacuole-containing heterotrophic bacterium [27]. The cyanobacteria were clearly shown to be the main source of polybrominated compounds, suggesting that the compound is not produced by the sponge itself [27].

Interestingly, the group of Agarwal and Moore finally provided proof of the origin of many PBDEs in 2014 [22]. They identified the biosynthesis pathway of polybrominated aromatic compounds in *Pseudoalteromonas* ssp. from *Dysidea* sp. extracts, which will later be reviewed in detail (see Section 5. Biosynthesis).

## 4. Chemical Diversity and Classifications

**Figure 1:**
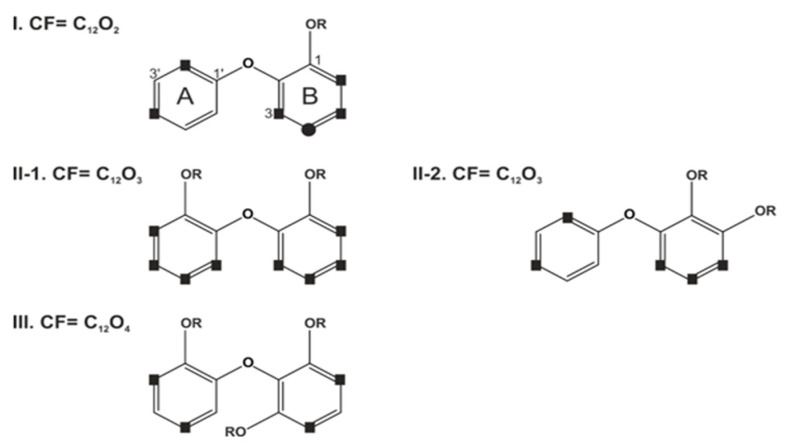
Structure types of sponge-derived O-PHDEs based on core formulas and substitution pattern. Possible halogenation sites: square = Br, circle = Br or Cl. Core formula (CF) based on diphenyl ether and attached oxygen atoms. R=H or CH_3_ [28].

After specifying the different sponge sources and their associated symbionts, compounds isolated from assumed sponge-bacteria extracts had to be classified. The first approach was published by Calcul et al. in 2009 [28]. They termed PBDEs more generally applicable ′polyhalogenated diphenyl ether′ (PHDE) and classified them into four structural types (I, II-1, II-2 and III) based on their core formulas shown in Figure 1 [28]. The current work will shortly review and adapt to this nomenclature in the following. The structure types (I, II-1, II-2, and III) differ in their core formula (CF) (number of oxygen atoms), substituents, and substitution patterns.

Only structure type I includes chlorine and bromine, although the inclusion of chlorine atoms is less common [28]. In addition to the discrimination based on the number of oxygen atoms, the substitution variations can be divided into further subtypes. All reported permutations of hydroxyl, halogens, and proton substitutions for rings A and B from known sponge-derived oxy-polyhalogenated diphenyl ether (O-PHDEs) are reviewed from known sponge-derived oxy-polyhalogenated diphenyl ether (O-PHDEs) in Calcul et al., 2009 [28]. Atoms of ring A are labeled with C1′–C6′, and those of ring B with C1–C6. The authors also review NMR (^1^H and ^13^C) data trends at protonated positions of ring A and B from sponge-derived O-PHDEs. They assumed that the OH at position C1 of ring B is biogenetically conserved [28], which was proven later by Agarwal et al. [22].

## 5. Biosynthesis

As mentioned above, it was thought until 2014 that PBDEs are abundant at all trophic levels of marine eukarya. However, neither bromophenol monomers nor OH-BDEs had been shown to be produced (only isolated) from marine bacterial sources. Sponge-associated symbiotic marine cyanobacteria were only presumed as producers of OH-BDEs [22,27]. Nevertheless, the only report of a synthesis pathway of polybrominated aromatic compounds with marine bacteria as a source of OH-BDEs with a solid genetic basis has been provided by Agarwal and Moore in 2014 [22]. The group classified OH-BDEs based on two distinct chemical signatures, the *para*- and *ortho*-coupling with respect to the hydroxy group [22].

They established experimentally tractable marine bacterial sources for the production of PBDEs and determined the genetic basis for their production by sequencing the genomes of *P.luteoviolacea* 2ta16 and *P.phenolica* O-BC30. Analysis of the genomes led to identification of the biosynthetic gene locus for brominated marine pyrroles/phenols (*bmp*) in both bacteria [22]. They investigated the genomes of both bacteria for proline dehydrogenase (PltE) and pyrrole halogenase (PltA), both enzymes used in pyoluteorin biosynthesis. Two homologs of the genes encoding for pltE and pltA (*bmp3* and *bmp2*) were found within genes encoding an acyl carrier protein (ACP)-thioesterase (TE) di-domain protein (*bmp1*), proline adenyltransferase (*bmp4*), flavin-dependent oxygenase (*bmp5*), chorismate lyase (*bmp6*), a cytochrome P450 (CYP450, *bmp7*), a carboxymuconolactone decarboxylase homolog (*bmp8*), ferredoxin (*bmp9*), and ferredoxin reductase (*bmp10*) [22]. Bmp8 is also known as a dehalogenating enzyme, which can remove Br residues from the ring at certain positions, rendering these available for dimerization [54]. An extensive review by Agarwal and colleagues from 2017 summarizes the mechanisms of halogenation and dehalogenation by these enzymes [54].

**Figure 2:**
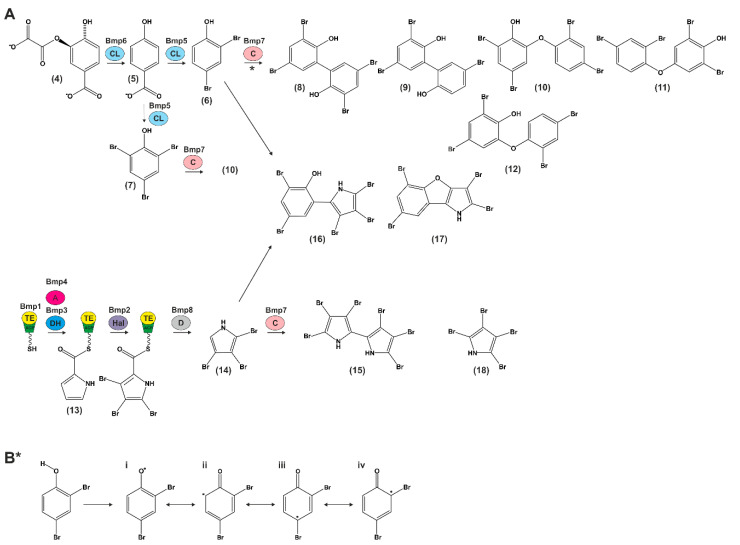
Bi-modular scheme for the biosynthesis of polybrominated marine natural products by the bmp pathway [22]. (**A**) Chorismate (**4**) converted to (**5**) by chorismate lyase (CL) Bmp6 and further to (**6**) and (**7**) by flavin-dependent halogenase Bmp5. The CYP coupling (C) enzyme Bmp7 generates different polybrominated biphenyls via the proposed steps in (B*), such as (**8**)–(**11**). Bromopyrroles are derived from L-proline, its acylation to the ACP domain (TE-ACP) of Bmp1 by proline adenyltransferase. (A) Bmp4 initiates its oxidation by the flavin-dependent dehydrogenase (DH) Bmp3. Followed by tribromination by the flavin-dependent halogenase (Hal) Bmp2. The TE domain of Bmp1 catalyzes the offloading of a carboxylic acid intermediate, which is decarboxylated (D) by carboxymuconolactone decarboxylase homolog Bmp8 to (**14**). (**14**) can be dimerized by Bmp7 to generate (**15**) and (**18**), or with (**6**) to generate heterodimers such as (**16**) and (**17**). (B*) Proposed steps for radical generation, rearrangement and coupling of (**6**) by Bmp7 to generate biphenyls and OH-BDEs. Adapted and modified from [22].

The *bmp* gene cluster is the first biosynthesis pathway shown to contribute to the production of polybrominated marine products (Figure 2A).

The biosynthetic pathway described in the following is derived from Agarwal and Moore and will be explained in brief [22] (Figure 2A). As an initiator molecule, chorismate (**4**) is converted to 4-hydroxybenzoic acid (4-HBA) (**5**) by chorismate lyase (Bmp6) and further to 2,4-dibromophenol (**6**) and 2,4,6-tribromophenol (**7**) by flavin-dependent halogenase (Bmp5). Bmp5 is the only known flavin-dependent halogenase that does not rely on a flavin reductase to regenerate the cofactor (single-component flavin-dependent halogenase) [54]. CYP450 coupling enzyme (Bmp7) generates a set of diverse polybrominated biphenyls (3,3′,5,5′-tetrabromo-2,2′-biphenyldiol (**8**), 3,5,5′-tribromo-2,2′-biphenyldiol (**9**)) and OH-BDEs from 2,4-dibromophenol (**6**) and 2,4,6-tribromophenol (**7**) (2′-OH-BDE-68 (**10**), 2,6-dibromo-4-(2,4-dibromophenoxy)phenol (**11**), 4,6-dibromo-2-(2,4-dibromophenoxy)phenol (**12**)). Bromopyrroles derive from L-proline (**13**), and the acylation of L-proline to the ACP domain of Bmp1 via proline adenyltransferase (Bmp4) initiates its oxidation by the flavin-dependent dehydrogenase (Bmp3) and tribromination by the flavin-dependent halogenase (Bmp2). The TE domain of Bmp1 catalyzes the offloading of a carboxylic acid intermediate, which is decarboxylated by carboxymuconolactone decarboxylase homolog Bmp8 to 2,3,4-tribromopyrrole (**14**). Further on, this compound can be homodimerized by Bmp7 to generate hexabromo-2,2′-bipyrrole (**15**) or heterodimerized with 2,4-dibromophenol to generate bromophenol-bromopyrrole pentabromopseudilin (**16**) and 2,3,5,7-tetrabromobenzofuro[3,2-*b*]pyrrole (**17**). The formation of 2,3,4,5-tetrabromopyrrole (**18**) was also observed during the heterodimerization of 2,3,4-tribromopyrrole.

Proposed steps for radical generation, rearrangement (i–iv), and coupling of 2,4-dibromophenol by Bmp7 to generate biphenyls and OH-BDEs are shown in Figure 2B* (i–iv) [22].

The group of Agarwal and Moore also showed that the *bmp* pathway can synthesize dibenzo-*p*-dioxins by employing different phenolic initiator molecules. They further diversified the structural classes of diphenyl ethers accessed by the pathway [44]. The authors proposed ideas of how the biosynthesis of dibenzo-*p*-dioxins could be performed.

On the one hand, biosynthesis of dibenzo-*p*-dioxins and di-OH-BDEs from 4-HBA (**5**) and 2,4-dibromophenols (**6**) would require hydroxylation of OH-BDEs to generate di-OH-BDEs, followed by an intramolecular cyclization to generate dibenzo-*p*-dioxins [44]. On the other hand, heteromeric coupling of bromocatechol and bromophenol monomers would generate the requisite phenolic skeletons of dibenzo-*p*-dioxins and di-OH-BDEs [44]. The authors assumed the heteromeric coupling to be more evident because the rates of nonspecific hydroxylation for OH-BDEs have been reported to be too low to support bioaccumulation and subsequent isolation of di-OH-BDEs and dibenzo-*p*-dioxins [55]. Also, bromoresorcinols and bromocatechols have been isolated from marine sources [37], supporting the hypothesis that marine bacteria could use phenolic molecules (dissimilar from 4-HBA), initiating biosynthesis of the polybrominated compounds [44]. The authors assumed that bromocatechols could derive from decarboxylative bromination of 3,4-dihydrobenzoic acid, analogous to the synthesis of 2,4-dibromophenol (**6**) from 4-HBA (**5**) catalyzed by the flavin-dependent halogenase Bmp5 [44]. 

Calcul et al., reviewed in 2009 that more than 43 O-PHDEs are derived from sponge-cyanobacterium associations [28]. Based on their classification, only bioactive compounds found in the literature will be separated into groups, and their effects will be reviewed below.

## 6. Bioactivity

### 6.1. Antibacterial, Antifungal, Antiviral Bioactivity

Antimicrobial activity of seven PBDEs against the Gram-positive bacterium *Bacillus subtilis* and the phytopathogenic fungus *Cladosporium cucumerinum* was first shown in 1997 [40]. Furthermore, a comprehensive study about isolated and synthetic PBDE derivatives by Hanif et al. revealed that many of the metabolites showed potent antibacterial activity against *Bacillus subtilis* [56]. In a more recent analysis, PBDEs were isolated from *Dysidea granulosa* and displayed antibacterial activity against methicillin-resistant *Staphylococcus aureus* (MRSA) and methicillin-sensitive *Staphylococcus aureus* (MSSA), *Escherichia coli* O157:H7, and *Salmonella* [35]. Another group investigated extracts of *Lamellodysidea* sp. and two collections of *D.granulosa* for their activity against the Gram-positive bacteria *Bacillus subtilis* and drug-susceptible and drug-resistant strains of *Staphylococcus aureus* and *Enterococcus faecium;* Gram-negative bacteria (*E.coli*); and against fungi (*C.albicans*). They tested 14 PBDEs for their antimicrobial activities against this panel of bacteria and fungi, and most of the compounds showed strong antimicrobial activity with low- to sub-µg/mL minimum inhibitory concentrations (MIC) [36]. The antifungal capacity of PBDEs against *Aspergillus* and *Candida* spp. was also investigated. Strikingly, one compound was active against both species in vitro and seemed to affect the fungal cell membrane primarily. The assumed mechanism was binding to sterols, thereby causing higher membrane permeability, inhibition of ergosterol synthesis, or an effect on membrane enzymes [48]. A recent publication by Ki et al., [57] isolated eight known brominated diphenyl ethers and a new tribromoiododiphenyl ether from the genus *Arenosclera* (belonging to *Callyspongiidae* family). Some of the compounds showed also antibacterial activities against *B.subtilis*, *S.aureus*, *K.pneumoniae,* and *E.coli* [57].

Besides their antimicrobial activity, PBDEs were also investigated for their antiviral potency. For example, 6-OH-BDE-47 (**19**) was identified to be an inhibitor of hepatitis C virus non-structural protein 3 (NS3 helicase) in a FRET-based high-throughput screening [45]. The group screened 17 PBDEs and related compounds on their NS3 ATPase inhibition capacity and found that the phenolic hydroxyl group has an important effect on the inhibitory activity [45]. Moreover, PBDEs were identified to target and inhibit the promotor region of hepatitis B virus (HBV) as well as the HBV production in HepG2.2.15.7 cells (human hepatocyte carcinoma) in a dose-dependent manner [58].

### 6.2. Synthetically Produced PBDEs

The marine environment consists of naturally produced PBDEs that structurally resemble synthetic brominated flame retardants (BFRs) [59]. Environmental toxicologists investigated the bioactivity of BFR-PBDEs, which, after the nomenclature, belong to polybrominated diphenyl ethers and are their synthetic analogs. The available publications on BFR-PBDEs focus on PBDEs in the food chain, air, soil, sediments, or consumer products, and on bioaccumulation in animals and even in humans. However, they mostly do so without considering a natural source for PBDEs. Furthermore, it has to be noted that many research papers investigated the toxicity of the most abundant synthetically produced BFR-PBDEs, neglecting the lower toxicity of naturally derived PBDEs with a lower level of bromination. Thus, all data deriving from these two sites of research have to be interpreted with caution.

In 2014, a biosynthesis pathway in bacteria was identified to be primarily responsible for the bioaccumulation in marine organisms. On the other hand, BFR-PBDEs can be synthesized and were found to be globally dispersed throughout the environment (reviewed by [60]), which is thought to be another source for bioaccumulation.

Laboratory studies showed the transformation from PBDEs to OH-PBDEs in fish, rat, and human cell culture [61,62,63,64,65]. Thus, there is considerable interest in the origin of OH- and MeO-PBDEs along with serious concerns about their bioactivity pattern based on the chemical structures.

### 6.3. Distinction of Naturally Produced PBDEs from Synthetic BFR-PBDEs

BFR-PBDEs are synthetic compounds used as additives to restrict fire and flames, and their mechanism of action is based on the thermally labile carbon-bromine bond. Thermal energy releases bromine radicals that intercept carbon radicals to decrease flames, simultaneously reducing heat and carbon monoxide production [66,67]. There are 209 possible congeners divided into 10 congener groups from mono- to deca-BDE, which are numbered according to the system developed by the International Union of Pure and Applied Chemistry [68,69]. They are commercially available in three technical mixtures as penta-, octa-, and deca-brominated diphenyl ethers. These PBDEs are also integrated in polymer matrices, where they are known to disperse from (reviewed in [70]). There is no information available on how much incineration of trash and leaching from landfills contributes to the environmental contamination and accumulation in organisms [70]. Concerning bioaccumulation in humans, BFR-PBDEs have been found in blood, serum, breast milk, adipose tissue, placental tissue, and in the brain. Also, prenatal transfer to embryo and fetus has been observed [67,71,72,73,74,75,76].

**Figure 3:**
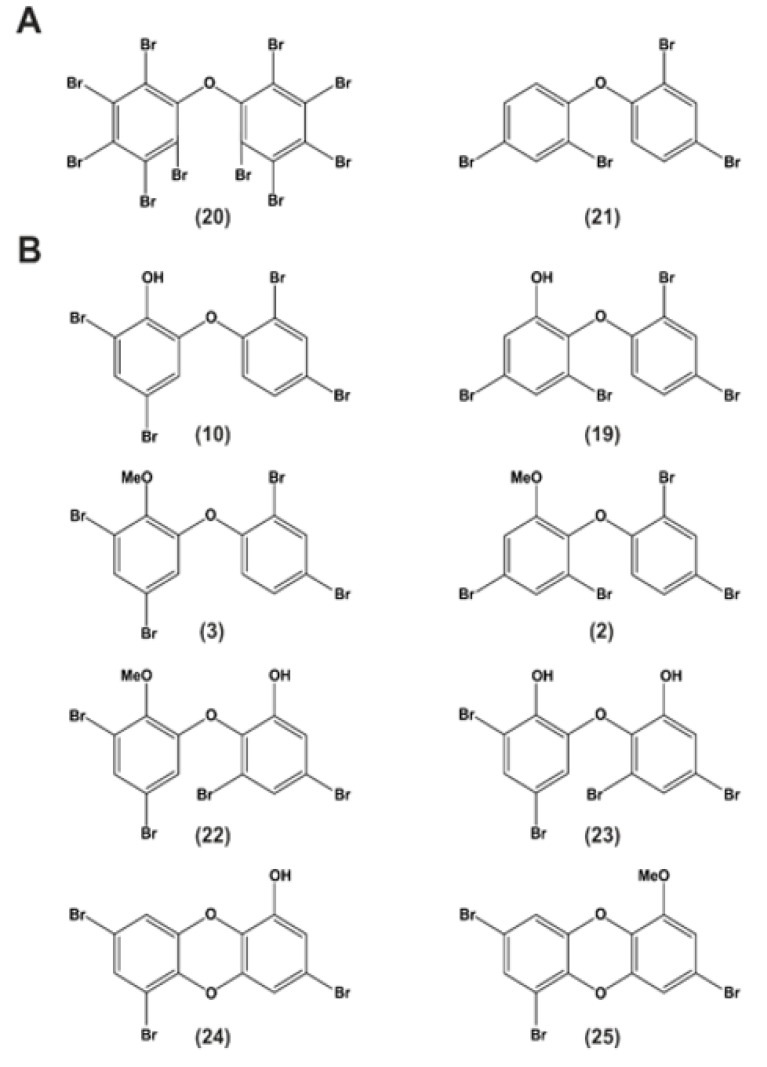
Synthetic and naturally occurring polyhalogenated molecules. Structural similarity between (**A**) synthetic and (**B**) naturally produced marine halogenated phenolic toxins and pollutants (MeO = methoxy-group) (adapted and modified from Agarwal et al., 2017).

Synthetically produced PBDEs do not exhibit the additional hydroxyl or methoxyl moieties like their natural analogs. Deca-BDE/BDE-209 (**20**) or BDE-47 (**21**) are depicted as examples in Figure 3A [59].

Naturally produced OH-PBDEs such as 2-OH-BDE-68 (**10**) or 6-OH-BDE-47 (**19**) and their methoxylated forms 2-MeO-BDE-68 (**3**) and 6-MeO-BDE-47 (**2**) are ubiquitous natural products (Figure 3B) [59]. In addition to OH-PBDEs, sponges also harbor methoxylated derivatives of dihydroxylated polybrominated diphenyl ethers (di-OH-BDEs), such as 2-MeO-6-OH-BDE-68 (**22**), which is thought to derive from 2,6′-OH-BDE-68 (**23**) and polybrominated dioxins, such as spongiadioxin C (**24**) or its methoxylated form (**25**) by methylation [59,77]. Agarwal and co-workers suggest that polybrominated natural products derived from sponges—amongst other natural sources—bioaccumulate in the marine food web with the potential to accumulate in humans [59].

In general, synthetic PBDEs exhibit low acute toxicity with an oral LD_50_ of >5 g/kg but upon chronic exposure, liver, kidney, and thyroid gland were the organs to suffer most from toxicity [74]. Observed effects of bioaccumulation in higher animals were later linked to endocrine disruption and neurotoxicity. Research in these fields expanded in the recent years and will be reviewed in the following part.

## 7. Bioactivity of Synthetic BFR-PBDEs

### 7.1. Thyrotoxicity

Synthetic PBDEs were found to compete with the binding of the thyroid hormone thyroxine (T_4_) (**26**) to its receptor transthyretin (TTR) upon metabolic conversion within rat liver microsomes (suggesting an important role for hydroxylation) [78]. With respect to the diversity of PBDEs, it was shown that OH-PBDEs are structurally more similar to thyroid hormones and have even a stronger bonding capacity to TTR than T_4_ [78,79,80,81,82]. Cao et al. investigated the binding of 14 diversely structured PBDE hydroxylates to thyroid hormone transport proteins and focused on the structure-mediated binding [81]. By molecular docking, they showed that all OH-PBDEs fit into the established TTR binding pocket.

**Figure 4:**
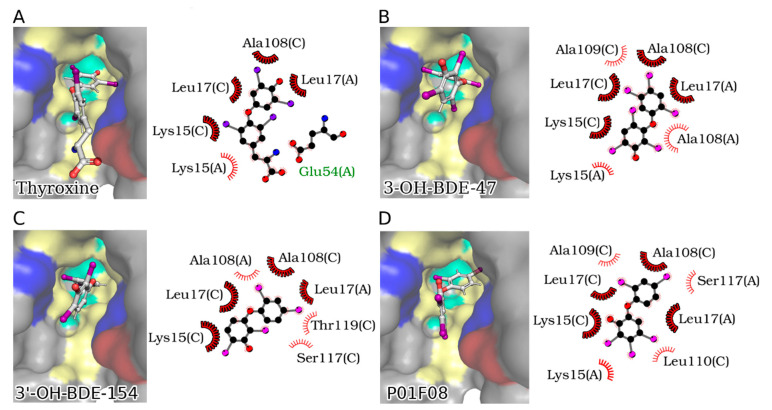
TTR-mediated thyrotoxicity of PBDEs. Crystal structure of TTR bound to T_4_ (PDB ID: 5CR1) (**A**) and docked 3-OH-BDE-47 (**B**), 3′-OH-BDE-154 (**C**), and P01F08 (**D**). For each figure, the left panel shows the accessible surface of the binding pocket. The hydrophobic surface is highlighted in yellow, the polar in cyan, the positively charged in blue, and the negatively charged in red. The right panel shows a schematic 2D representation of the interactions between the ligand and the residues of the binding pocket. Carbon, nitrogen, oxygen, iodine, and bromine atoms are represented in black, blue, red, purple, and magenta, respectively. Single-letter chain identifiers are present next to each residue. Hydrophobic contacts are shown as red splines curves. Hydrophobic contact common for all ligands are highlighted in bold.

Using a site-specific fluorescence probe, the binding of hydroxylated PBDEs to thyroid hormone transport proteins was shown by Ren and Guo [83] with a focus on thyroxine-binding globulin (TBG) and TTR. By conducting our own docking experiments using P01F08 (Figure 4D) and the two most effective OH-PBDEs characterized by Ren and Guo [83] (Figure 4B,C), we found that the diphenyl ether backbone adopts an orientation similar to T_4_ in the TTR binding site (Figure 4D). In all cases, the OH group points to the solvent, as expected, and similar to the carboxylate group in T_4_ (Figure 4A). Given the abundance of hydrophobic residues in the binding pocket, likely affine interactions can be formed with the bromo substituents of PBDEs, which are oriented very similarly to the iodo substituents of T_4_. Eleven OH-PBDEs with different bromination levels and different hydroxylation positions were assessed for their binding affinity with TBG and TTR, respectively, indicating that the binding affinity generally increased with the number of bromines but also that the position of the hydroxyl group was of importance [83]. Ren and Guo concluded that PBDEs have the potential to disrupt thyroid homeostasis by competitive binding with thyroxine transport proteins [83] (3-OH-BDE-47 (**27**) and 3′-OH-BDE-154 (**28**) exhibited the strongest effects).

### 7.2. Neurotoxicity

Extensive research about PBDE-mediated neurotoxicity on the (developing) nervous system was performed by Dingemans and colleagues [80,84,85]. Animal studies with different BFR-PBDEs indicated that pre- and postnatal exposure may cause long-lasting behavioral alterations especially affecting the motor activity and cognitive behavior. Animal studies in mice revealed that brain development is most sensitive to exposure to PBDEs in the first 2 weeks after birth, where the synaptogenesis and myelination take place. As a link to human brain development, it can be concluded that these processes take place in the last trimester of pregnancy and extend into early childhood [80,86,87,88].

Neurochemical changes were also observed. Neonatal exposure to BDE-47 (Figure 3) (**21**) was found to impair long-term potentiation in the mouse hippocampus (a form of synaptic plasticity associated with memory) [89]. Similar findings were observed in rat dentate gyrus in vivo when exposed to the fully brominated BDE-209/Deca-BDE (Figure 3) (**20**) during different developmental stages [90]. Another BFR-PBDE, BDE-99 (**29**), increased the activity of the glutamate-nitric-oxide-cyclin guanosine monophosphate pathway in the rat cerebellum [91].

Summarizing, the effects of BFR-PBDEs on the brain were observed in the hippocampus, cortex, striatum, and cerebellum. Alterations of protein levels involved in synaptic plasticity and brain development were also detected (reviewed in [80]). These effects were seen upon treatment with tetra- and penta-BDEs or octa- through deca-BDEs [80].

Referring to cell viability, several in vitro studies showed tetra- and penta-BDEs to induce apoptosis in primary neurons or neuronal cell lines, which was assumed to result from oxidative stress [92,93,94,95,96]. Besides effects on cell viability, also cell differentiation, migration, and neuronal signaling were impaired upon PBDE exposure in several studies (reviewed in [80]). Taken together, it was postulated that tetra- or penta-brominated PBDEs affect all levels of neurotransmission, such as compromising the presynaptic neurotransmitter homeostasis up to their release to postsynaptic receptors. Although OH-PBDEs have a higher potency than their parent congeners, the main molecular target of (OH-)PBDEs remains elusive [80].

### 7.3. Other Effects

Upon exposure to BDE-209/Deca-BDE (Figure 3) (**20**), an increased incidence of hepatocellular carcinomas and thyroid adenomas has been observed in rodents. Also, prenatal exposure to BDE-99 (**29**) was found to reduce sperm counts in adult rats. Thus, PBDEs also influence the reproductive capacity. In addition, some tetra-BDEs and OH-tetra-BDEs caused genotoxicity by DNA damage through reactive oxygen species (ROS), leading to replication blockage and subsequent chromosomal breaks in chicken DT40 cell lines [97]. Some BFR-PBDEs have been reported to induce CYP1A and CYP2B (DE-71), CYP2B and CYP3A (BDE-47, -99, -153 (**30**)), and many others (reviewed in detail in [74,80]) (CYP= Cytochrome P450 protein family, involved in the metabolism of xenobiotics in the body). A detailed table of in vitro studies about selected PBDEs (BDE-47 (**21**), 6-OH-BDE-47 (**19**), and 6-MeO-BDE-47 (**2**)) is listed in [80].

## 8. Naturally Produced PBDEs

### 8.1. Bioactivity of Natural PBDEs

**Figure 5:**
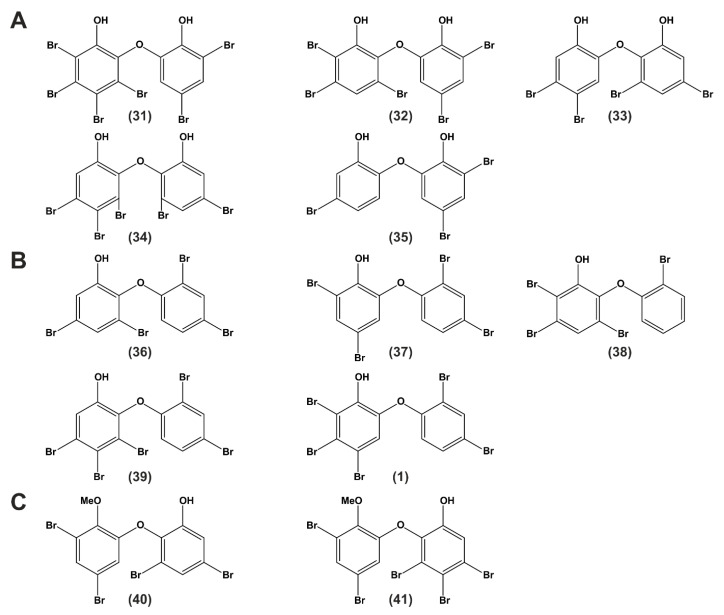
Structures of compounds with reported bioactivity in terms of apoptosis, cytotoxicity, or cancer. (**A**) Published di-OH-PBDEs, (**B**) Published OH-PBDEs, and (**C**) Published MeO-OH-PBDEs.

The literature was screened for available bioactivity data of naturally produced and isolated PBDEs, di-OH-BDEs and MeO-PBDEs. Data referring to bioactivity in terms of apoptosis, cancer, and cytotoxicity of selected compounds are summarized in Table 2 and Table 3 and the corresponding structures are listed in Figure 5.

### 8.2. Apoptosis and Cancer

There is only a limited amount of data available on natural PBDEs and their anticancer activity. The first investigations on possible therapeutic application appeared in literature in 1995, when Fu and colleagues tested extracts and pure compounds isolated from *Dysidea* sp. sponges and assayed them against five different enzymes, four of which are relevant to anticancer drug discovery. Fifteen compounds were tested for their inhibitory capacity on 15-lipoxygenase (LO), inosine monophosphate dehydrogenase (IMPDH), guanosine monophosphate synthetase (GMPS), protein tyrosine kinase (PTK), or matrix metalloprotease (MMP) [37]. The analysis of marine-derived brominated diphenyl ethers as inhibitors of human platelet 12-LO, human reticulocyte 15-LO, and soybean 15-LO was performed by Segraves et al. [98]. The group found that some PBDEs are able to reduce the active site iron of the enzyme, which was however assumed not to be responsible for the inhibitory potential. Moreover, they showed that dioxins like (**24**) are more potent inhibitors of human reticulocyte 15-LO than (**32**) [98].

PBDEs isolated from the marine sponge *Phyllospongia dendyi* were tested for their inhibitory activity on the assembly of microtubule proteins because the microtubule system is an important target for the development of anticancer drugs [49]. Three compounds (**31**)–(**33**) had an effect on the assembly of microtubule proteins at 29.6, 33.5, and 20.9 µM (IC_50_), respectively [49].

Another screening study analyzed five PBDEs from *Phyllospongia dendyi*. The compounds with two phenol units were reported to inhibit the assembly of microtubule proteins in vitro [49] and showed a weak inhibition in colony formation of V79 (Chinese hamster) cells and an increase in IL-8 production [51] (see Figure 5A (**31**), Figure 5 (**32**) and (**33**)). IL-8 expression has been detected in numerous cancers and was suggested to be a factor in tumor progression and metastasis [51].

Polybrominated diphenyl ethers from sponges of the *Dysidea* genus were also investigated concerning their potential to inhibit the Tie2 kinase [43]. Tie2 kinase is an enzyme supporting angiogenesis in tumor growth and survival, and thus represents a potential target for antitumor therapy. Xu et al. suggested that the two investigated PBDEs (see Figure 5A (**34**) and (**35**)) display dissimilar structures to a known Tie2 kinase inhibitor [99]. Moreover, the hydrophobic moiety was assumed to interact with a hydrophobic pocket close to the binding site and a hydrophilic moiety participating in hydrogen bonding to the ATP binding site of the kinase, which are necessary for the inhibitory activity [43]. The results presented by Xu and co-workers indicate that the brominated phenol system might bind more efficiently to the ATP binding site because the diphenyl ethers investigated in that study showed even better inhibitory activity than the control (Tie2 kinase inhibitor) [43].

The inhibitory potential of PBDEs on α-D-galactosidase was also investigated and shown by Utkina et al. [100]. This enzyme is important for the hydrolysis of non-digestible polysaccharides in the human gut. Another study by de la Fuente and colleagues showed the inhibitory properties of PBDEs on aldose reductase, which plays a role in diabetes mellitus, identifying a marine natural PBDE as new human aldolase reductase inhibitor with an IC_50_ of 6.4 µM (see Supplementary Figure S1 (**31**)) [101].

In 2008, the group of Zhang et al. aimed to find a natural product-based protein phosphorylation inhibitor using a hyphae formation inhibition (HFI) assay in Streptomyces 85E to screen for compounds that target general serine/threonine and/or tyrosine kinase activities [38]. Because aerial hyphae formation requires protein kinase activity, they suggested that natural products, which inhibit this formation, might also block the proliferation of cancer cells. They analyzed nine compounds isolated from *Dysidea* sp. The compounds (**36**) and (**38**) (Figure 5B) showed inhibitory activity in the HFI assay. All compounds were also tested on MCF-7 human breast cancer cells, and the IC_50_ values were determined. (**36**) (Figure 5B) was most active with an IC_50_ of 2.84 µM and further evaluated in cell cycle analysis. A cell cycle arrest with 80% of the cells in G1-phase could be observed in serum-free medium, whereas only 60% of the cells were in G1 in serum-containing medium. A concomitant increase in the number of cells in S-phase was observed, indicating that serum treatment reversed the G1 arrest. The effect could be blocked when cells were exposed to (**36**) at 10 µM concentration prior to serum treatment, suggesting that the compound blocks a signal transduction pathway necessary for initiation of DNA synthesis in MCF-7 cells. The group did not detect any cell death within a 2–10 µM range. However, at higher concentrations, cytotoxicity occurred. The authors assumed that small structural changes such as the bromination position and number of substituents might affect the activity of the compounds. Moreover, they postulate that the biological activity decreases with an increase in bromine substituents in the A ring [38].

A compound screening of 2240 compounds by a FRET-assay aimed at identifying drugs that might disrupt the interaction between Mcl-1 and Bak [28]. Mcl-1 and Bak belong to the Bcl-2 family. Blocking the binding of antiapoptotic Mcl-1 to proapoptotic Bak can initiate the endogenous suicide program [102]. The group focused on extracts from *Dysidea granulosa* and semi pure compounds from *Dysidea* (*Lamellodysidea*) *herbacea*, which exhibited activity in a primary FRET screen. More than 42 known O-PHDEs exist, which are derived from sponge-cyanobacterium associations [28]. Only compound (**36**), (**37**), and (**39**) (Figure 5B) showed significant IC_50_ values <10 µg/mL in the Mcl-1/Bak FRET screen [28].

Arai et al. specifically searched for selective growth inhibitors against cancer cells that had adapted their metabolism to nutrient starvation. They identified two PBDEs isolated from *Dysidea* sp. to display anti-proliferative activity against the pancreatic carcinoma cell line PANC-1 under glucose-starvation conditions ((**39**) and (**36**) (Figure 5B)) [39]. The authors assumed that the anti-proliferative efficacy results from the inhibition of complex II enzyme in the mitochondria. Thus, the compounds would be potential candidates for further development as anticancer drugs [39].

Recently, the first data concerning the effect of a naturally derived PBDE on human primary healthy and malignant cells were published by Mayer et al. [17]. Two compounds were isolated from the marine sponge *Dysidea* sp., termed P01F08 (**1**) and P01F03 (**37**) (Figure 5B). The group treated T cell leukemia cells (Jurkat J16) and B cell lymphoma cells (Ramos) with both compounds and could show a strong antiproliferative activity, also for acute promyelitic cells (HL-60) and acute monocytic leukemia cells (THP-1) [17]. Furthermore, these compounds were tested for potential cytotoxic side effects on healthy human peripheral blood mononuclear cells (PBMNCs) using suspension culture and colony-forming unit assays. This revealed a therapeutic window compared to data from primary malignant cells derived from patients with acute myeloid leukemia (AML). In particular, P01F08 (**1**) showed a 3.2-fold lower IC_50_ value in primary leukemic cells compared to the PBMNCs of healthy donors. Thus, these data demonstrate the therapeutic relevance of polybrominated diphenyl ethers for patients with a cancerous disease such as AML [17].

Besides the potential effects on cancer cells, the general cytotoxicity of naturally derived PBDEs on non-transformed cells must to be taken into account. Relevant data on cytotoxicity and the tested concentrations on diverse test systems are reviewed in the following.

### 8.3. Cytotoxicity on Non-Transformed Cells

The first study investigating the cytotoxicity of PBDEs extracted from *Dysidea herbacea* was published in 2005, where compound (**40**) (Figure 5C) was tested in the human cell line Hep2. Cells were treated with the minimum inhibitory concentration (MIC), and metabolic activity was assayed. A decrease of 25% of the metabolic activity of the cells was noted after 1 h of exposure. This compound was also tested on human red blood cells (RBCs), where it caused hemolysis in 10% of RBCs after 1 h of exposure. Treating with five-fold MIC (39 µg/mL) increased hemolysis to almost 70% [48].

In a second study, PBDEs originally isolated from *Lamellodysidea herbacea* and modified by synthetical derivatization were tested for their cytotoxic potential against benign NBT-T2 rat bladder epithelial cells and showed moderate to weak cytotoxicity [56]. IC_50_ values of compound (**37**) and compound (**35**) (Figure 5A,B) were found to be 2.8 and 8.5 µg/mL, respectively [56].

Another study tested 14 polybrominated diphenyl ethers isolated from *Dysidea granulosa* and *Lamellodysidea* sp. in a monkey kidney cell line (Bsc-1). Some compounds showed toxicity against the kidney cell line Bsc-1 with IC_50_ values between 7 and 35 µg/mL. The A rings of these compounds lack a hydroxy group and contain bromine atoms *ortho* and *para* to the ether compared to the other tested substances. The lack of the hydroxy group on ring A and/or bromine substitution pattern led to increased cytotoxicity with compound (**36**) (Figure 5B) being the most cytotoxic one [36].

Up to now, these are the only available cytotoxicity data of PBDEs isolated and naturally derived from marine (sponge) origin on murine, monkey, or human test systems.

## 9. P01F08—Structural Information

**Figure 6:**
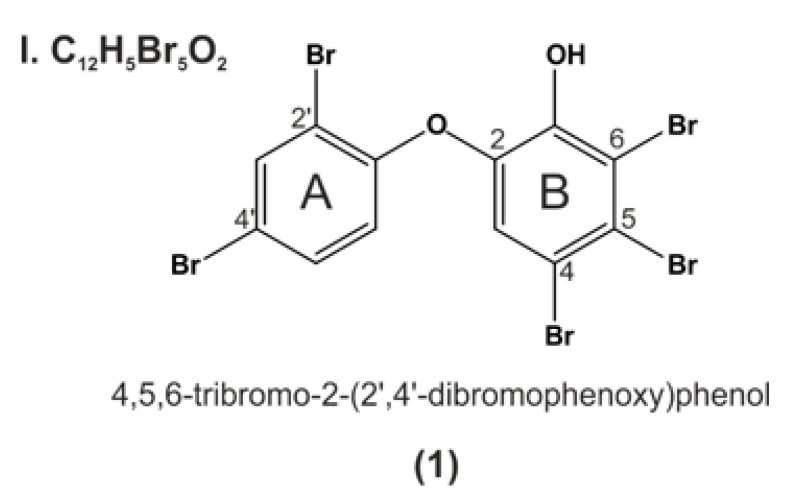
P01F08 (**1**) belongs to class I according to the nomenclature of Calcul et al., 2009. The A ring comprises two bromine substituents in 2′ and 4′ position, linked by an ether bond to ring B comprising three bromine substituents at position 4,5,6, and a hydroxy group in ortho position.

When comparing all data (IUPAC names or structures) about PBDEs isolated from natural sources, the first isolation of P01F08 (**1**) (Figure 6) was described in 1981 by Carté and Faulkner [47]. They isolated the compound of interest from *Dysidea herbacea* and named it 2-(2′,4′-dibromophenoxy)-4,5,6-tribromophenol (structure (2) in Carté and Faulkner., 1981) [47].

^13^C NMR data for compound P01F08 (compound 3 in Fu and Schmitz.,1996) were first published in 1996 [18]. This group was one of the first investigating the anticancer potential of the PBDEs, respectively, and identified P01F08 (**1**) (compound **14** in Fu et al., 1995) to inhibit 15-LO at IC_50_ values of 7.4 µM and inosine monophosphate dehydrogenase at EC_50_ of 4.4 µM. Seventy-one percent inhibition at 50 µM was detected for guanosine monophosphate synthetase. No inhibition of protein tyrosine kinases or matrix metalloproteases was observed for this compound [37].

In another recent publication, P01F08 was isolated from collections of *Dysidea granulosa* and tested for cytotoxicity in a monkey kidney cell line (Bsc-1) and a human colorectal tumor cell line (HCT-116). P01F08 was one of the compounds that was cytotoxic against the Bsc-1 cells with an IC_50_ of 15 µg/mL. Interestingly, the compound also inhibited the growth of the following bacteria: *S.aureus* ATCC 29213, *S. aureus* ATCC 43300, *E.faecium* ATCC 29212, and *E.faecium* ATCC 51299 with minimum inhibitory concentrations between 3.7 and 0.4 µg/mL [36].

As reviewed in the previous part Apoptosis and Cancer, Mayer and colleagues published results identifying P01F08 as a promising anticancer drug, which showed a therapeutic window caused by its lower cytotoxicity against PBMNCs of healthy donors compared to malignant primary leukemic cells of AML patients [17]. Based on these interesting data, research with P01F08 was conducted leading to novel data and a mechanistic hypothesis regarding its activity, which will be presented in the following part.

## 10. Results and Discussion P01F08

As reviewed above, PBDEs show a wide range of bioactivity depending on their structural differences, such as diverse bromination patterns and location of hydroxy groups. Recently, we tested P01F08 (**1**) in different cell lines, identifying a therapeutic window enabling the usage of P01F08 against AML [17]. The elucidation of the mechanistic pathway of cell death induction is indispensable for the prospective of P01F08 as anticancer drug. We therefore aimed (i) to clarify if P01F08′s cytotoxicity is limited to T cell leukemia (Jurkat) or B cell lymphoma (Ramos) cells; (ii) compare the efficacy of apoptosis induction in both cell types; and (iii) investigate which apoptosis signaling pathway is induced (intrinsic or extrinsic).

### 10.1. P01F08 is Highly Cytotoxic in Burkitt′s Lymphoma B Cells (Ramos) and Acute T Cell Leukemia (Jurkat)

**Figure 7:**
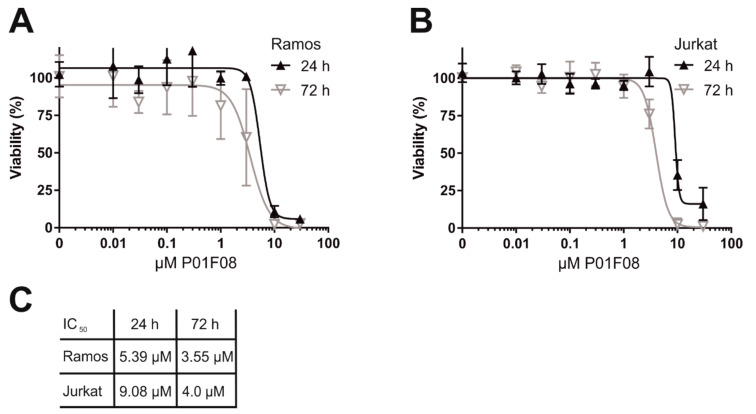
P01F08 acts highly cytotoxic in acute T cell leukemia (Jurkat) and Burkitt′s lymphoma B (Ramos) cells. Cytotoxicity in Ramos (**A**) or Jurkat (**B**) cells was determined after the indicated incubation periods using alamarblue viability assay. (**C**) Overview of the resulting IC_50_ values in the individual cell lines at the respective incubation times. All experiments were performed in triplicates; the values were normalized to DMSO (0.1% *v*/*v*; negative control). Error bars = SD of three independent experiments performed in triplicates.

To determine the cytotoxic potential of P01F08, we performed a cytotoxicity assay (Resazurin reduction assay (alamarblue assay)) in acute T cell leukemia (Jurkat) and B cell lymphoma cells (Ramos) after 24 and 72 h of incubation in a concentration-dependent manner (Figure 7). P01F08 acts highly cytotoxic in Ramos cells (Figure 7A) (IC_50_ 24 h: 5.39 µM) and to a lesser extent in Jurkat cells (Figure 7B) (IC_50_ 24 h: 9.08 µM). For both cell lines, the cytotoxicity increases with the duration of incubation (Figure 7C).

### 10.2. P01F08 is a Potent Inducer of Apoptosis in Ramos and Jurkat Cells with Short Latency and Rapid Kinetics Especially in Ramos Cells

Apoptosis is defined as a genetically programmed cell death pathway. In mammalian cells, it can be activated by at least two major signaling routes, the extrinsic death receptor-mediated pathway and the intrinsic mitochondrial pathway, which both depend on the activation of intracellular cysteine proteases (caspases).

The external pathway initiates apoptosis via ligation of death receptors, such as CD95, TRAIL-R1, and TRAIL-R2 with their respective ligands. Upon binding of the trimeric ligand, cytoplasmic adaptor proteins such as FADD are recruited to the death receptor by the mutual interaction of the death domains of both death receptors and FADD. FADD subsequently recruits initiator procaspase-8 via a mutual interaction of their death effector domains. On formation of this death-inducing signaling complex (DISC), procaspase-8 is activated by dimerization and autoproteolytic cleavage [103].

The intrinsic apoptosis pathway is activated by cellular stress such as DNA-damage (e.g., irradiation or anticancer drugs), toxins, hypoxia, viral infections, or radicals, and results in the release of cytochrome *c* from the mitochondrion. The mitochondrial cytochrome *c* release is mediated via proapoptotic Bcl-2 proteins (such as Bax or Bak), which could be blocked by antiapoptotic Bcl-2 proteins (such as Bcl-2, Bcl-xL or Mcl-1) [102]. Since activation of the mitochondrial apoptosis pathway is the major mechanism of radio- and chemotherapy, tumor cells can acquire resistance by inactivating this cell death route—e.g., via overexpression of antiapoptotic Bcl-2 proteins [102]. In the cytosol, cytochrome *c* acts as a second messenger and binds together with deoxyadenosine triphosphate (dATP) to the adapter protein Apaf-1. Apaf-1 subsequently oligomerizes and recruits procaspase-9 via mutual interaction of their caspase recruitment domains (CARDs). In this high molecular weight complex, termed apoptosome, the initiator procaspase-9 is subsequently activated [103].

The initiator caspases of both apoptosis pathways proteolytically activate downstream located effector caspases (such as caspase-3). Subsequently, both signaling pathways induce cell death via the effector caspase-mediated cleavage of respective apoptosis substrates [103]. Thus, activation of caspase-3 as the most prominent effector caspase leads to proteolytic processing of several substrates, such as poly (ADP-ribose) polymerase 1 (PARP1), which is inactivated upon proteolytic cleavage. In addition, caspase-3 activates caspase-activated DNase (CAD) by cleaving the corresponding inhibitor of caspase-activated DNase (iCAD), leading to the fragmentation of chromosomal DNA [104,105].

**Figure 8:**
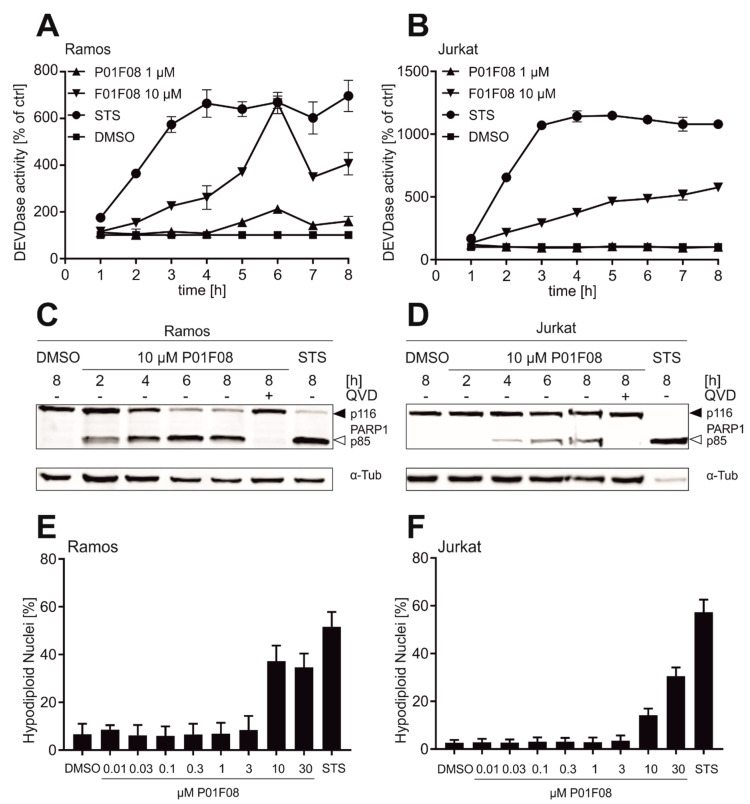
P01F08 is a potent inducer of apoptosis in leukemia and lymphoma cells with short latency and rapid kinetics especially in Ramos (lymphoma) cells. Ramos (**A**) and Jurkat (**B**) cells were treated with a high concentration of P01F08 (10 µM) or staurosporine (STS; 2.5 µM; positive control) for the induction of apoptosis for 8 h. Subsequently, DEVDase activity as a surrogate marker for caspase-3 activity was determined via measurement of the fluorescence of the profluorescent caspase-3 substrate DEVD-AMC in a micro-spectrophotometer. The slope of the linear range of fluorescence increase served as a measure for DEVDase activity. The DMSO control values were set to 100% and the normalized relative fold induction was calculated as described in Materials & Methods. (**A**) and (**B**) are representative for three independent experiments; mean and SD of triplicates are depicted. (**C**) and (**D**) show representative immunoblots of three independent experiments of cleavage of the caspase-3 substrate poly(ADP-ribose) polymerase 1 (PARP1; full-length 116 kDa, cleaved form 85 kDa) as an indicator for apoptotic cell death in Ramos cells (**C**) and Jurkat cells (**D**). Cells were treated with indicated concentrations of P01F08 (10 µM), DMSO (0.1% *v*/*v*), and STS (2.5 µM) for the indicated incubation times alone or with pre-treatment (30 min) of the pan-caspase inhibitor QVD (10 µM). anti-Tubulin (α-Tub) served as a loading control. (**E**) and (**F**) Apoptosis-related DNA degradation was detected after 24 h incubation via flowcytometric measurement of propidium iodide stained hypodiploid nuclei in (**E**) Ramos and (**F**) Jurkat cells. Mean and SD of three independent experiments performed in triplicates are depicted.

To further compare the efficacy of apoptosis induction in both cell types, we performed caspase-3-activity assays (Figure 8). Ramos (Figure 8A) and Jurkat (Figure 8B) cells were treated with 1 or 10 µM P01F08 and caspase-3 activity was monitored in an 8 h kinetics. In Ramos cells, caspase-3 activity can be detected as early as 2 to 3 h after 10 µM P01F08 treatment and peaks after 6 h. In Jurkat cells, caspase-3 activity steadily increases upon treatment with 10 µM P01F08. For both cell lines, almost no caspase-3 activity was observed when treated with 1 µM P01F08. Similar to the cytotoxicity measurements, Ramos cells seem to be slightly more susceptible to treatment with P01F08 than Jurkat cells (lower IC_50_ and higher caspase-3 activity).

Due to the higher caspase-3 activity at 10 µM, both cell lines were treated with 10 µM of P01F08 for further experiments, and the cleavage of PARP1 by caspase-3 was monitored in an 8 h kinetics (Figure 8). In Ramos cells (Figure 8C), P01F08 rapidly induces PARP1 cleavage within the first 2 h of incubation. In Jurkat cells (Figure 8D), P01F08 induces delayed PARP1 cleavage starting after 4 h of incubation. Additionally, it was checked whether this event is only mediated due to the induction of caspase activation. Therefore, cells were pre-incubated with the pan-caspase inhibitor quinoline-val-asp-difluorophenoxymethylketone (QVD-OPH). For both cell lines, PARP1 cleavage can be prohibited upon pre-treatment with QVD. Thus, the induction of cell death is obviously caspase-dependent.

To further assess P01F08′s ability to induce apoptosis, we next determined the amount of apoptotic hypodiploid nuclei by propidium iodide (PI) staining according to the method of Nicoletti et al. [106], which detects the amount of DNA fragmentation (Figure 8). P01F08 caused a concentration-dependent increase in hypodiploid nuclei in both cell lines, with Ramos cells (Figure 8E) being again more susceptible than Jurkat cells (Figure 8F).

### 10.3. P01F08 Induces Bcl-2 Dependent Apoptosis

**Figure 9:**
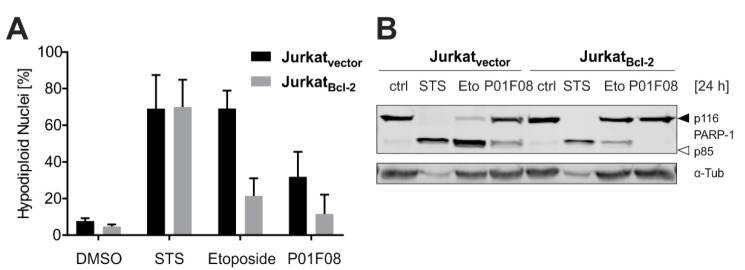
P01F08 induces Bcl-2 dependent apoptosis. Jurkat cells overexpressing Bcl-2 and corresponding vector control cells were treated with 2.5 µM staurosporine (STS), 50 µM Etoposide, and 10 µM P01F08 for 24 h. (**A**) Apoptosis-related DNA degradation was detected via flowcytometric measurement of propidium iodide stained hypodiploid nuclei. Mean and SD of three independent experiments performed in triplicates are depicted. (**B**) Representative immunoblot of three independent experiments of cleavage of the caspase-3 substrate poly(ADP-ribose) polymerase 1 (PARP1; full-length 116 kDa, cleaved form 85 kDa) as an indicator for apoptotic cell death. anti-Tubulin (α-Tub) served as a loading control.

After demonstrating that P01F08 induces caspase-mediated apoptosis in both cell lines but to a different extent, we next investigated whether it triggers the intrinsic apoptotic mitochondrial pathway. Based on the detailed literature available, we know that polybrominated diphenyl ether derivatives have a wide bioactivity pattern, targeting also many bacteria species. If a compound targets prokaryotic and eukaryotic organisms, it is very likely that mitochondria are affected. Consequently, we wanted to investigate whether apoptosis induction by P01F08 is mediated via the mitochondrial death pathway. For this purpose, we used Jurkat cells overexpressing antiapoptotic Bcl-2 or the corresponding empty vector control and determined the amount of hypodiploid nuclei in Nicoletti assay after 24 h (Figure 9A). The cells were treated with the respective controls, staurosporine (STS; 2.5 µM) and etoposide (50 µM) (Figure 9A,B).

Staurosporine (STS) is a widely used potent apoptotic stimulus that, similar to DNA-damaging anticancer drugs, can activate the mitochondrial death pathway independent of external death receptor signaling. In addition, STS is also proficient in inducing apoptosis via an alternative route independent of the mitochondrial apoptosis pathway [107]. Etoposide is widely used as an anticancer drug that induces DNA damage by inhibiting topoisomerase II, resulting in the induction of the intrinsic apoptosis cascade [108]. Thus, the induction of apoptosis via STS is not blocked by overexpression of Bcl-2 due to the activation of an alternative pathway, whereas intrinsic apoptosis induced by etoposide is inhibited upon Bcl-2 overexpression in Nicoletti and Western Blot analysis. Regarding P01F08, the compound generally induced lower levels of apoptosis in Jurkat cells, but, interestingly apoptosis was completely blocked by overexpression of Bcl-2. This clearly proves that apoptosis induction with P01F08 activates the intrinsic mitochondrial pathway of apoptosis (Figure 9B).

In summary, the general cytotoxicity of P01F08 appeared to be time- and concentration-dependent. Also, P01F08 was shown to be more cytotoxic in Ramos cells than Jurkat cells. By contrast, it induced caspase-dependent apoptosis in both cell lines but with dissimilar potency and latency. Apoptosis induced with P01F08 could be blocked in Bcl-2 overexpressing cells, suggesting that this compound targets the mitochondrial death pathway. Interestingly, when monitoring the Bcl-2 expression levels in wild type Ramos and Jurkat cells (i.e., without Bcl-2 overexpression), it was evident that Ramos cells do not express endogenous Bcl-2 (data not shown). Thus, this might be a general reason for Ramos cells being more susceptible to cytotoxic stimuli because they lack the antiapoptotic function of Bcl-2. Further investigations could show if other mechanisms, such as increased cytotoxic activity under glucose-starved conditions with altered Akt signaling [39] or disturbed Ca^2+^ homeostasis [85] mediating potential mitochondrial stress, are responsible for the induction of intrinsic apoptosis in cancer cells.

The following structure activity relationship (SAR) summarizes and interprets bioactivity data of three natural and synthetical analogs of P01F08. The findings serve as a basis for further research on PBDEs and therapeutic applications.

## 11. Structure–Activity Relationship Analysis of P01F08

With this SAR analysis, we aim to determine the chemical group(s) responsible for evoking (a) special biological effect(s). This allows follow-up investigations of the effects or enhancing the biological activity by changing the chemical structure. An intense screening of the PBDE literature was performed, distinguishing synthetic PBDE-related research from natural PBDE-related research, where the exact mechanism behind the SAR of PBDEs remains poorly understood.

**Figure 10:**
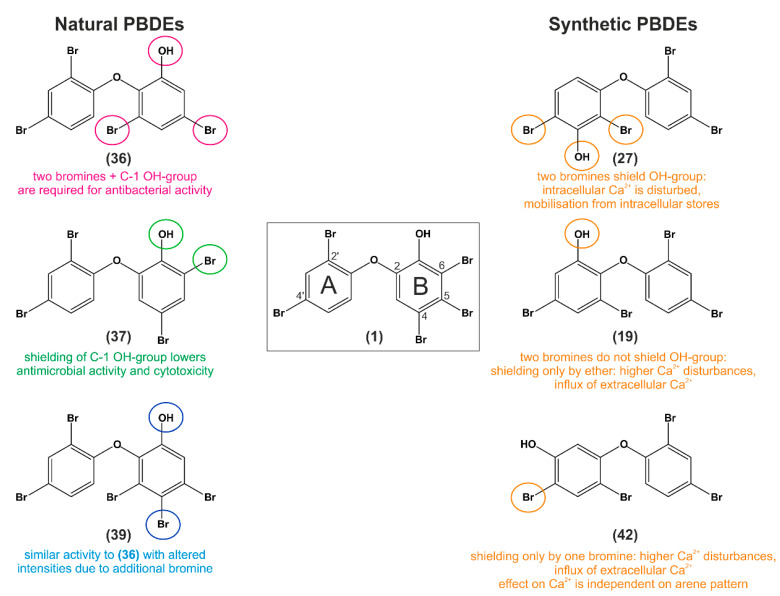
Selected natural [**(36**), (**37**), (**39**)] and synthetic [(**27**), (**19**), (**42**)] PBDEs for the structure–activity relationship analysis compared to P01F08. Each, three natural and three synthetic compounds were chosen on equal position and number of bromine substituents at the A-ring, equal position of the ether connecting ring A and B, equal position of phenol-group at B-ring, and at least two bromine substituents have to be at the same position at the B ring similar to P01F08. For each compound, the published SAR conclusions of the corresponding literature source have been added. When investigating natural PBDEs, the researcher focused on the number of bromines and position of OH-group with their influence on bioactivity, respectively (shown in pink for (**36**), green for (**37**) and blue for (**39**)). With investigating synthetic PBDEs, we selected SAR conclusions from Dingemans et al. [80,84,85,89] and highlighted the corresponding substituents mediating different bioactivities in orange for (**27**), (**19**), and (**42**). For detailed description of the experimental setups used in the literature, refer to the text.

The three naturally derived PBDEs most similar to P01F08 were chosen based on the following parameters: (1.) Equal position and number of bromine substituents at the A ring (C-2′ and C-4′ positions) (2.) Equal position of the ether oxygen between the A and B rings (C-1′ - C-2). (3.) Equal position of the phenol group at the B ring (B C-3). (4.) At least two bromine substituents at the B ring must be present and, if possible, at the same position as in P01F08 (Figure 10).

The three most similar, synthetic PBDEs to P01F08 were chosen based on identical parameters but neglecting the position of the phenol group in the context of neighboring bromine substituents (Figure 10).

Comparing the naturally derived PBDEs to P01F08, it has to be noted that all naturally derived PBDEs comprise the phenol group and the two bromines at the A ring due to their common biosynthesis pathway [22]. The first compound (**36**) (Figure 10) has only one overlapping bromine substitution at ring B position C-5 compared to P01F08 and an additional at C-3 (it lacks bromines at ring B C-4 and C-6). This compound was shown to be active against *S.aureus* (0.042–0.08 µg/mL), *E.faecium* (1.2 µg/mL) and *E.coli* (3.1 µg/mL) with toxicity to Bsc-1 cells (7 µg/mL) [36]. The authors assumed that the lack of an additional hydroxy group at the A ring and/or the bromine substitution pattern leads to increased cytotoxicity. Based on their SAR analysis, they postulated that ring B needs two bromine atoms and a C-1′ hydroxy group for antibacterial activity. Moreover, the presence of two phenolic hydroxy groups at C-1′ and C-2 in PBDEs decreases the cytotoxicity along with a loss of activity against the Gram-negative bacterium *E.coli* [36]. Similar cytotoxicity data for this compound against several Gram-positive and Gram-negative bacteria were published by Sun et al. [35]. The authors concluded that bromination changes electron density and hydroxyl radical reactions. These could influence antimicrobial bioactivities and thereby contribute to bacterial cell death. They hinted at oxidative damage as a potential cellular death pathway, which has to be elucidated [35]. This compound was also analyzed on its antiproliferative activity using an MCF-7 human breast cancer cell line and was the most active with an IC_50_ of 2.84 µM. The authors linked a decrease in biological activity with an increase in the number of bromine substituents in the A ring (here: B ring) [38]. In a Mcl-1 FRET assay, this compound was the most important, showing inhibitory activity with an IC_50_ of 2.1 µg/mL for the interaction of the antiapoptotic Bcl-2 member Mcl-1 with proapoptotic Bak [28].

In terms of SAR analyses, the authors suggest that two hydrophobic moieties, one interacting with a hydrophobic pocket close to the binding site and the other participating in hydrogen bond to the ATP binding site of kinases, are necessary for inhibitory activity [43]. For the tie2 kinase, a quinone flanked by a hydrophobic group had been suggested to be efficient for binding to the kinase [109]. This is contrary to the results presented by Xu et al., which indicate that a brominated phenol system may bind more efficiently to the ATP binding site of the tie2 kinase [43]. In another study, this compound (**36**) was investigated for its antiproliferative activity against human pancreatic carcinoma (PANC-1) cells [39]. It showed inhibitory activity with an IC_50_ of 3.8 µM under glucose-deprived conditions and no activity under normal glucose conditions [39].

The second compound (**37**) (Figure 10) has two overlapping bromine substitutions compared to P01F08 at ring B positions C-4 and C-6, but lacks the bromine of P01F08 at position C-5. This compound was present in most of the studies reviewed above, was less active against *S.aureus* (0.78–0.19 µg/mL), more active against *E.faecium* (0.8 µg/mL), not active against *E.coli* (>100 µg/mL), and almost not cytotoxic against Bsc-1 cells (32 µg/mL) [36]. In terms of its differential activity towards Gram-positive and Gram-negative bacteria (Table 1 and Table 2, DG-2 in [35]), it was the compound with minor antimicrobial activity compared to the two others. These data are contrary to a publication by Ki et al., 2019, which postulated compound (**37**) to be the most active against all four tested bacteria (*B.subtilis*, *S.aureus*, *K.pneumoniae*, *E.coli*) compared to (**36**) [57]. Compound (**37**) was described to be less antiproliferative (compared to (**36**)) in MCF-7 cells (IC_50_ 8.9 µM ± 7.41) [34], it showed no (<10 µg/mL) inhibitory activity in the Mcl-1 FRET assay [28]. Compared to the Tie2 inhibitor, (**37**) was less active (6.2 µM) [43]. Recently, (**37**) was shown in Mayer et al. (as P01F03) to be less cytotoxic in Jurkat cells, HL-60, and TP-1 cells after 24 h of incubation [17].

A third compound (**39**) (Figure 10) will be compared additionally because it also has two overlapping bromine substitutions at ring B position C-4 and C-5 compared to P01F08. It lacks a bromine at C-6, but has an additional bromine at C-3. In general, this compound is the most analogous naturally derived PBDE to P01F08, because it fulfills all criteria 1.–4.) and has in sum, the same number of bromine substitutions. It is less cytotoxic against *S.aureus* (0.14–0.015 µg/mL), more cytotoxic against *E.faecium* (0.4 µg/mL), and less cytotoxic to *E.coli* (12.5 µg/mL), with a comparable cytotoxicity against the Bsc-1 cells (8.8 µg/mL) [36]. Compared to the previous compound (**37**), (**39**) was comparably inefficient (<10 µg/mL) in inhibiting the interaction between Mcl-1 and Bak in the Mcl-1 FRET assay [28]. It was published in Sun et al., that this compound exhibits a lower activity against several Gram-negative bacteria: *Salmonella* sp., *E.coli*, and *Pseudomonas* ([35] see publication Table 2, D-1). It can be noted that it was still active against Gram-positive bacteria, but to a lesser extent than the other compounds tested in that screening [35]. Regarding its antimicrobial activity, data about its antifungal capacity were published by Sionov et al., this compound was found to be active against *A.fumigatus* and *C.albicans* at MIC concentrations of 7.81 and 15.62 µg/mL [48]. (**39**) was also investigated in a recent study by Arai et al., regarding its antiproliferative activity against PANC-1 cells under glucose-starved and general culture conditions. It showed no antiproliferative activity under general culture conditions, whereas it showed inhibitory activity with an IC_50_ of 2.1 µM under glucose deficient conditions ((**39**) was more active than its analog (**36**)) [39].

Interestingly, the group also investigated the effect of (**39**) on the mitochondrial electron transport chain and compared it to antimycin A, which is known to be an inhibitor of complex III. Using the Mito Check Complex Activity Assay Kit (Cayman Chemical), they showed that (**39**) strongly inhibits complexes II and III with IC_50_ values of 6.4 nM and 0.86 µM and assumed that complex II may represent the primary target [39]. 

Taken together, all three naturally derived PBDEs ((**36**), (**37**) and (**39**)) reviewed in this part showed different patterns of antimicrobial, antiproliferative, and cytotoxic activity. Based on these data, it can be assumed that a shielding of the phenolic group (besides the shielding by the ether binding), as for (**37**), lowers the potency of PBDEs in terms of broader antimicrobial activity. It can be postulated that this bromine substitution at ring B position C-6 lowers the activity of the compound.

Regarding the SAR of the other bromines at ring B position C-3, C-4, or C-5, it can only be assumed that (**36**) and (**39**) both are bioactive against bacteria and cancer cells in the lower µM range, and both compounds contain bromines at C-3 and C-5. When comparing (**36**) to P01F08 (**1**), it has to be noted that a compound with only two bromines exhibited less cytotoxicity and antineoplastic activity than P01F08 (**1**) with three bromines. Whether an additional bromine at ring B (e.g., at C-4) decreases or increases bioactivity cannot be postulated. Therefore, a test system with all four compounds should be set up to screen for antimicrobial and antiproliferative capacity in Gram-positive and Gram-negative bacteria, in fungi, and for several cancerous cell lines.

As reviewed above, synthetic PBDEs are a group of brominated flame retardants with various toxic effects on the test systems (see Section 7. Bioactivity of Synthetic BFR-PBDEs). These BFR-PBDEs were shown to affect spontaneous behavior in rodents and learning efficacy. Moreover, they have been detected in humans. Especially neurotoxicity studies and endocrine studies demonstrate the importance of unraveling the full mechanism of action of these substances. It is important to notice that CYP2B6 has been found to predominantly transform (BFR-)PBDEs to OH-PBDEs and MeO-PBDEs in humans [110]. This has been shown for the most popular BFR-PBDE, BDE-47 (**21**), which was transformed into six congeners of OH-PBDEs [110]. The fact that the BFR-PBDEs can be oxidatively metabolized into even more toxic congeners for the organism was extensively reviewed by Dingemans et al. [85], who provided a detailed overview about the neurotoxicity and their (in)direct effects of parent compounds compared to hydroxylated PBDEs on the (developing) nervous system [80] (involves many BDEs (-47,-49,-99,-100,-153,-183,-203,-206,-209, a commercial penta-BDE product (DE-71), and 6-MeO-BDE-47 (**2**) and 6-OH-BDE-47 (**19**)).

As mentioned above, the three synthetic PBDEs most similar to P01F08 were chosen, neglecting the position of the phenol group in the context of neighboring bromine substituents (Figure 10). Three possible parameters of compound properties will be reviewed: the influence of the shielding of the phenolic hydroxy group, the arene substitution pattern (*ortho*, *meta* and *para*), and the planarity of PBDEs compared to their congeners (e.g., polychlorinated biphenyls (PCBs)).

The first synthetic BFR-PBDE is 3-OH-BDE-47 (**27**), where the position of the OH group is *meta* to the ether and two bromine substituents shield this OH group [84]. In mechanistic investigations where all synthetic BFR-PBDEs ((**27**), (**19**), and (**42**)) were compared to each other, (**27**) disturbed intracellular Ca^2+^ homeostasis (source: endoplasmic reticulum (ER) and mitochondria) [84]. Similarly, the second synthetic BFR-PBDE 6-OH-BDE-47 (**19**), where the position of the OH group is *ortho* to the ether, has the same effect on Ca^2+^ homeostasis as (**27**) [84]. For (**19**), the OH group is not shielded by any neighboring substituent. Interestingly, this compound seems to be similar to naturally-derived (**36**) when comparing the structures, but they were published and reviewed in the literature with different IUPAC names. Therefore, the accumulated data are sorted in this article depending on the context, if it is a synthetically ordered BFR-PBDE ((**27**) = synthetic PBDE) or naturally derived PBDE from a marine source ((**36**) = natural PBDE). The third synthetic BFR-PBDE in the comparison is (**42**) = 5-OH-BDE47, where the position of the OH group is also *meta* to the ether and shielded by only one bromine [84].

Regarding SAR analyses, the group of Dingemans postulated after detailed research that OH-PBDEs, in which the OH group was shielded on only one side by either bromine or a phenyl ring, induce a more pronounced disturbance of Ca^2+^ homeostasis by different sources [80,84,85]. It has to be noted that the influence of the shielding of the OH group was independent of its possible position (*ortho*, *meta,* or *para*) on the PBDE molecule [84]. Moreover, the group detected no or mild effects on cell viability for the investigated OH-PBDEs, indicating that the observed effects were not impaired by cytotoxicity in the corresponding concentrations [84]. When discussing the influence of the arene substitution pattern, an analysis of De la Fuente et al. [101] should be considered (mainly di-OH-PBDEs). The group synthesized and analyzed PBDEs on their human aldose reductase inhibitory activities, with an extensive structure–activity analysis demonstrating the importance of free hydroxy groups in enzyme binding [101].

Another SAR study investigated the binding of 11 OH-PBDEs to thyroid hormone transport proteins [83]. They found that TTR binding potency was associated with the degree of bromination, and hydroxylation in position 3-*meta* or 2-*para* increased the RP (=binding affinity of the chemical with the transport protein in comparison to T_4_) with an increasing number of bromines [83]. The group investigated (**27**), (**19**), and (**42**) on these parameters. These three OH-PBDEs exhibit the same level of bromination (tetra-) but different OH positions—the highest binding potency was observed for (**27**)**,** followed by (**19**) and (**42**). The same was observed for dibromo and tribromo compounds, where the one with 3-*meta* OH exhibited stronger TTR binding potency than the 2-*para* hydroxylated compound [83]. Referring to potential SAR hypotheses, the group postulated that two chemical functionalities affect the binding potency of OH-PBDEs, the degree of bromination, and the position of the hydroxylation. An increase in the number of bromines goes along with an increase of K_ow_/cLogP (n-Octanol/Water Partition Coefficient). cLogP is a parameter to measure the relationship between water solubility (hydrophilicity) and fat solubility (lipophilicity) of a compound. The value is larger than zero if a compound is more lipophilic (such as n-octanol) and smaller than zero if it is more hydrophilic. Thus, if an increase of bromination correlates with an increase in clogP, the compounds are more lipophilic with higher bromination number, meaning here that the increase correlates with stronger hydrophobic interactions with the protein [83]. A study that also discussed the topic of hydrophobicity was performed by Seagraves et al. [98]. The authors assumed that the potency of 15-LO inhibition correlates with increasing bromination and an increase of hydrophobicity depending on position of the bromine and/or an increase in the size of the molecule [98].

A recent analysis by Utkina et al. [100] supports the importance of hydrophobicity for the related effects of PBDEs. They showed that an increase in bromination correlates with potency in inhibiting α-D-galactosidase and an increase of hydrophobicity (clogP values), respectively (for BFR-PBDE OH-BDE-47 (**19**) and BDE-153 (**39**)) [100]. The group demonstrated for di-OH-PBDEs that an additional hydroxy group enhanced potency in inhibiting α-D-galactosidase, whereas an additional methoxy group decreased the inhibition potency [100].

Regarding the concentration dependency of the effects, OH-PBDEs (BFR-PBDEs: (**27**), (**19**), and (**42**)) were found to increase basal [Ca^2+^]_i_ at high concentrations (5–20 µM) in chromaffin and pheochromocytoma (PC12) cells along with the inhibition of depolarization-evoked [Ca^2+^]_i_ [85]. This inhibition seemed to be more sensitive to increases in basal [Ca^2+^]_i_ by Ca^2+^ release from intracellular stores by (**27**) than to those due to influx of extracellular Ca^2+^ by (**19**) or (**42**) [85].

In sum, synthetic OH-PBDEs where the OH group was shielded on both sides by atomic groups (bromine atoms or aromatic rings), such as (**27**), had fewer effects than OH-PBDEs that shielded only at one side ((**19**) and (**42**)) [85]. This observation is concordant with the finding of Salam et al. that (**36**) (isolated from extracts of marine organisms, but structurally equal to (**19**)) was identified as an inhibitor of NS3 ATPase activity in a high-throughput fluorescence helicase assay based on FRET [45]. The group analyzed the SAR of different PBDEs and related compounds, postulating that the biphenyl ring, bromine, and phenolic hydroxy group on the benzene backbone are the essential groups mediating the inhibitory potency [45].

Concerning the influence of the planarity of these molecules, it has been demonstrated for *ortho* PCB congeners (but not for coplanar ones) that they alter Ca^2+^ homeostasis by inducing changes in the integrity of mitochondrial and ER membranes, which is accompanied by a decrease in the mitochondrial membrane potential and an accumulation of intracellular Ca^2+^ [111,112]. Additionally, it has been shown that PCB 47 (**43**) and PCB 52 (**44**), which are non-coplanar congeners, significantly compromised the plasma membrane integrity with an accumulation of intracellular Ca^2+^ [113]. The authors assumed that disruption of the membrane structure, either the plasma membrane or an organelle membrane, could cause the changes in ion permeability via voltage or ligand-gated channels or changes in the activity of enzymes bound to the membrane [113]. These nonspecific effects were thought to contribute also to the loss of Ca^2+^ sequestration, as presented in [111,113]. Referring to their thyroid toxicity, it has to be noted that the total OH-PBDE concentration in blood ranges from 0.012–0.48 nM [114], which is lower in comparison with total T_4_ concentrations (58–161 nM). Therefore a displacement of T_4_ from transport proteins by OH-PBDEs might be less likely [83].

This raises the question, what is the physiological significance of these investigations for environmental toxicology adjustments, and how likely is an impairment of the developing nervous system of children when exposed to PBDEs? Referring to the data presented by Dingemans et al., the median (21.96 ng/g lipids) and highest (899.1 ng/g lipids) concentration of total OH-PBDEs were observed in fetal plasma corresponding to approximately 0.4 and 17.4 nM in blood, respectively (the blood concentration is calculated from exposure values at a lipid weight adjusted basis (ng/g lipids)) [84]. The highest concentration observed in human blood would only be lower by two orders of magnitude than the lowest observed effective concentration (LOEC; 1 µM) for disturbing the Ca^2+^ homeostasis [84]. Nevertheless, the LOEC for increased Ca^2+^ fluctuations is lower (0.2 µM), which causes concerns that the margin of exposure is insufficient in some individual exposure situations [84].

When discussing the relevance of the comparison between synthetic and naturally derived PBDEs, it has to be taken into account that the differences and similarities in terms of biological activity for both groups are poorly understood. This article aimed to summarize data about polybrominated diphenyl ether derivatives of about the last 40 years of research, distinguishing especially the data of naturally derived PBDEs from data of synthetic PBDEs in terms of chemical properties and biological activity data. After extensively expounding different hypotheses about the SAR of these molecules, it can be assumed that if the mechanism of action is equal for both substance groups, it might be likelier mediated by the chemical properties of the compounds than by a direct compound-target interaction.

## 12. Materials and Methods

### 12.1. Molecular Modelling

The crystal structure of TTR in complex with thyroxine (PDB ID 5CR1) was selected to generate a receptor model for docking of PBDEs. Alternative sidechain configurations were removed using the Protein Preparation Wizard in Maestro. The bond order and possible protonation states of the selected ligands were assigned using LigPrep (LigPrep, Schrödinger LLC, New York, NY, USA). A cubic grid with a side length of 18 Å was centered around the thyroxine. Dockings were carried out using Glide-XP [115] implemented in Schrödinger’s Maestro Suite (Schrödinger LLC, New York, NY, USA) using default set-ups. 2D representations of the docked ligands were generated using LigPlot+ [116].

### 12.2. Compound P01F08

P01F08 was obtained from the compound library of Peter Proksh at the Institute for Pharmaceutical Biology and Biotechnology of the Heinrich Heine University Düsseldorf, and freshly prepared and dissolved in DMSO. Until use for the assays, the compound was kept at −20 °C in a temperature-controlled refrigerator.

### 12.3. Cell Lines and Cell Culture

Jurkat cells (#ACC-282) were obtained from DSMZ; Ramos cells were kindly provided by Michael Engelke (Institute of Cellular and Molecular Immunology, University Hospital Göttingen, Germany). Stable transfectants of Jurkat cells with Bcl-2 overexpression and corresponding empty vector control cells were kindly provided by Claus Belka [117] (Department of Radiation Oncology, University Hospital, LMU Munich, Germany). All cell lines were maintained at 5% CO_2_ at 37 °C and stable humidity in RPMI 1640 medium supplemented with 10% FCS, 100 U/mL penicillin, and 100 µg/mL streptomycin.

### 12.4. Reagents

Staurosporine (STS, #9300) was obtained from LC Laboratories (Woburn, MA, USA), *N*-(2-quinolyl)valyl-aspartyl-(2,6-difluorophenoxy)methyl ketone (QVD, #S7311) from Selleckchem (Houston, TX, USA) and Etoposide from BioVision (#1043-100).

### 12.5. Cytotoxicity Measurements

For the determination of cytotoxicity in Ramos and Jurkat cells, the resazurin reduction assay, which is also known as alamarBlue^®^ assay, was performed as previously described [118]. In short, cells were seeded at a specific density depending on the incubation time (24 h: 1 × 10^6^ cells/mL, 72 h: 0.2 × 10^6^ cells/mL), incubated with increasing compound concentrations, and after a specified treatment time, resazurin (Sigma, #R7017) was added to a final concentration of 40 µM. After 120 min of incubation, the fluorescence of resorufin (excitation (Ex): 535 nm, emission (Em): 590 nm) was measured with a microplate spectrophotometer (Synergy Mix platereader). DMSO (0.1% *v*/*v*) was used as negative control and staurosporine (2.5 µM) as positive control. Viability of control cells was set to 100% and all other values were normalized to the control. The reduction of resazurin to resorufin is proportional to aerobic respiration. Therefore, it serves as a measure for the cell viability and cytotoxicity of a tested compound.

### 12.6. Fluorimetric Analysis of Caspase-3 Activity (DEVDase Assay)

The caspase-3 activity assay was performed as described in [119]. Briefly, Ramos or Jurkat cells were seeded at a density of 1 × 10^6^ cells/mL in a 96-well plate, treated with the compound for depicted time durations (kinetics 0–8 h), harvested at 900 g, 5 min, at 4 °C, lysed on ice in lysis buffer containing 1 µg/mL leupeptin, 5 µg/mL aprotinin, and 1 µg/mL pepstatin. Cell lysates were transferred to a microplate and mixed with ice-cold reaction buffer containing the profluorescent caspase substrate Ac-DEVD-AMC (Biomol GmbH, Hamburg, Germany, #ABD-13402). The increase in DEVDase-dependent fluorescence was measured at Synergy Mix microplate reader at 37 °C for 120 min every 2 min (Ex 360 nm, Em 450 nm). The slope of the linear range of fluorescence increase over 120 min represents caspase-3 activity.

### 12.7. Immunoblotting

Cells were seeded at a density of 1 × 10^6^ cells/mL, treated as specified, and harvested by centrifugation (3000× *g*, 5 min) followed by freezing in liquid nitrogen. The cell pellets were thawed on ice, quick-frozen in liquid nitrogen, and defrozen three times, mixed with lysis buffer and lysed on ice for a further 30 min, accompanied by vortexing. Subsequently, centrifugation (13,300× *g*, 15 min) purified cell lysates from cell debris, and the protein concentration in the supernatant was determined with Bradford assay. The samples were diluted with sample buffer, and SDS-PAGE and Western Blot were conducted in accordance with standard workflows. Finally, target protein-specific primary antibodies (anti-PARP1 1:2000 (Enzo, #BML-SA250); anti-Tubulin 1:2000 (Sigma, #T5168); and fluorescence-coupled secondary antibodies (LI-COR Biosciences) were used for the detection of target proteins on PVDF membrane using LI-COR Odyssey^®^ imaging system.

### 12.8. Propidium Iodide (PI) Uptake (Nicoletti Assay)

Cells were seeded at a density of 1 × 10^6^ cells/mL in a 96-well plate, treated as specified with indicated concentrations of P01F08 and STS (2.5 µM) as a positive control. The nuclei of Ramos or Jurkat cells were prepared by lysing cells in hypotonic lysis buffer [1% sodium citrate, 0.1% Triton X-100, 50 µg/mL propidium iodide (PI)] for 120 min at 4 °C. After lysis, the PI fluorescence was measured via flow cytometry. PI binds stoichiometrically to DNA, thus, PI fluorescence mirrors DNA content of the prepared nuclei [106]. Data were extracted from FACS DIVA software and analyzed in FlowJo (Flow Cytometry Analysis Software). Hypodiploid nuclei were considered as apoptotic and shown is the percentage of hypodiploid nuclei of the whole nuclei population.

### 12.9. Replicates and Statistical Analysis

Experiments were replicated at least three times, and representative data are shown. Error bars indicate standard deviation.

## Figures and Tables

**Table 1:** List of sponge species associated with the production of PBDEs, corresponding isolation sources, and respective references.

Specimen	Isolation Sources and References
*Dysidea* sp.	Chuuk Atoll and Fiji (Indo-Pacific) [18,37]
	Federated States of Micronesia [38]
	Maumere, Indonesia [39]
	*P.luteoviolacea* 2ta16 (Florida Keys), *P.phenolica* O-BC30 (coast in Japan) [22]
	West Sumatra, Indonesia [40]
	Pelorus Island, Great Barrier Reef, Australia [41]
	Islands Tutuila and Ofu (Eastern Samoa) [42]
	Isolation from crude extract [43]
	Fiji ICBG program gave access to sponge sample [44]
*Dysidea* (*Lamellodysidea*) *herbacea*	Ujungkulon, Indonesia [19]
	Pocklington reef in Milne Bay, Papua New Guinea [28]
	Chuuk Atoll and Giji (Indo-Pacific) [37]
	West Sumatra, Indonesia [40]
	Okinawa Islands, Japan [45]
	US Territory of Guam [46]
	Iwayama Bay, Palau [47]
	Coast of Zanzibar [48]
	Palau [49]
*Dysidea granulosa*	Pocklington reef in Milne Bay, Papua New Guinea [28]
	Natural Product Depository
	Division of National Institutes of Health supplied the methanol extracts [35]
	Papua New Guinea and Palau Islands [36]
	Okinawa Islands, Japan [45]
	US Territory of Guam [46]
*Dysidea chlorea*	Iwayama Bay, Palau [47]
*Dysidea fragilis*	No source given [49,50]
*Dysidea arenaria*	Coast of Zanzibar [48]
*Phyllospongia dendyi*	Palau [49]
	Palau [51]
*Phyllospongia foliascens*	Iwayama Bay, Palau [47]

**Table 2:** Literature summary on bioactivity data of naturally derived and isolated PBDEs in relation to apoptosis and cancer. The literature about PBDEs was screened for data about bioactivity related to apoptosis or cancer cell lines. The corresponding publication is listed together with investigated compounds (structures are shown in Figure 5) and respective biological effects.

Publication	Structure in Figure 5	Effects Related to Apoptosis or Cancer Cell Lines
Fu et al., 1995	(**36**) **(37**) (**40**) (**32**) (**41**) (**34**) (**39**) (**1**) (**31**)	- Inhibition of 15-LO, IMPDH, GMPS, MMP, none against PTK
Liu et al., 2004	(**31**) (**32**) (**33**)	- Inhibited assembly of purified porcine brain microtubule proteins
Oda et al., 2005	(**31**) (**32**) (**34**)	- Weak inhibition of colony formation of V79 cells Increased IL-8 production
Xu et al., 2005	(**36**) (**37**)	- Inhibition of Tie2 kinase
Zhang et al., 2008	(**36**) (**38**)	- Inhibitory activity against hyphae formation as possible serine/threonine and/or tyrosine kinase inhibitors - Tested against MCF-7 cells and cytotoxic at low µM concentrations - Cell cycle arrest in MCF-7 cells
Calcul et al., 2009	(**36**) (**37**) (**39**)	- Significant IC_50_ values <10 µg/mL in the Mcl-1/Bak FRET screen
Arai et al., 2017	(**39**) (**36**)	- Antiproliferative activity against PANC-1 cells under glucose-starved conditions
Segraves et al., 2004	(**31**) (**32**)	- Inhibitory activity against human reticulocyte 15-LO: (**31**) IC_50_: 0.79 ±0.07 µM, (**32**) IC_50_: 2.2 ± 0.4 µM
Mayer et al., 2019	(**37**) (**1**)	- P01F03 (**37**): cytotoxic on Jurkat and Ramos cells in low µM range (1–2 µM), for THP-1, HL-60, PBMNCs, malignant patient cells in high µM range (19–31 µM) P01F08 (**1**): cytotoxic on Jurkat, Ramos, THP-1, HL-60, malignant patient cells in low µM range (1–6 µM), for PBMNCs in high µM range (19 µM) Induction of apoptosis in Jurkat and Ramos cells by both Caspase-3-activity in Jurkat and Ramos cells, no induction of caspase-3-activity in PBMNCs for P01F08

**Table 3:** Literature summary on bioactivity data of naturally derived and isolated PBDEs in relation to cytotoxicity on murine, monkey, or human cell lines. The literature on PBDEs was screened for data concerning bioactivity in murine, monkey, or human cell lines. The corresponding publication is listed together with most active compounds (structures are shown in Figure 5) and their respective biological effects.

Publication	Structure in Figure 5	Cytotoxic Effects of Naturally Derived PBDEs on Murine, Monkey or Human Test Systems
Sionov et al., 2005	(**40**)	- Human cell line Hep2 was treated with 3.9–31.5 µg/mL and metabolic activity was decreased after 1 h exposure 1 h exposure (7.8–39 µg/mL) caused hemolysis inhuman red blood cells
Hanif et al., 2007	(**41**) (**35**)	- Moderate to weak cytotoxicity against NBT-T2 cells (IC_50_ 2.8 µg/mL) Moderate to weak cytotoxicity against NBT-T2 cells (IC_50_ 8.5 µg/mL)
Liu et al., 2016	(**39**)	- Cytotoxicity against Bsc-1 cells (14 compounds with IC_50_ of 7–35 µg/mL); (39) was the most cytotoxic one (IC_50_ = 7 µg/mL)
Mayer et al., 2019	(**1**) (**37**)	- Cytotoxicity against PBMNCs low after 72 h IC_50_ of 19.62 µM Cytotoxicity against PBMNCs higher than P01F08 IC_50_ after 72 h 1.56- 3.01 µM

## Data Availability

Not applicable.

## Supplementary Materials

The following are available online, Figure S1: List of all compounds mentioned in text and figures with their corresponding Arabic numeral. The IUPAC name(s) of every compound, which is mentioned in the text and in this figure can be identified with its corresponding Arabic numeral in Supplementary Table S1.; Table S1: List of all compounds mentioned in text and figures with their abbreviations and IUPAC names, with a corresponding Arabic numeral. The structure of every compound, which is mentioned in text and in this table can be identified with its corresponding Arabic numeral in Supplementary Figure S1.
