# Supplementary material for: 40 Years of Research on Polybrominated Diphenyl Ethers (PBDEs)—A Historical Overview and Newest Data of a Promising Anticancer Drug
Source: Molecules. 2021 Feb 13;26(4):995. doi: 10.3390/molecules26040995 (PMC7918430; doi:10.3390/molecules26040995)
Supplement: Supplementary file 1 [file molecules-26-00995-s001.pdf]

Supplemental Material

Supplemental Table S1. List of all compounds mentioned in text and figures with their abbreviations and IUPAC names, with a corresponding Arabic numeral. The structure of every compound, which is mentioned in the text and in this table can be identified with its corresponding Arabic numeral in Supplemental Figure S1.

Arabic numeral.	**Compound abbreviations and IUPACs used in text or literature**
**(1)**	4,5,6-tribromo-2-(2′,4′-dibromophenoxy)phenol or P01F08
**(2)**	6-MeO-BDE-47
**(3)**	2-MeO-BDE-68
**(4)**	Chorismate
**(5)**	4-hydrobenzoic acid
**(6)**	2,4-dibromophenol
**(7)**	2,4,6-tribromophenol
**(8)**	3,3′,5,5′-tetrabromo-2,2′-biphenyldiol
**(9)**	3,5,5′-tribromo-2,2′-biphenyldiol
**(10)**	2′-OH-BDE-68
**(11)**	2,6-dibromo-4-(2,4-dibromophenoxy)phenol
**(12)**	4,6-dibromo-2-(2,4-dibromophenoxy)phenol
**(13)**	L-proline
**(14)**	2,3,4-tribromopyrrole
**(15)**	hexabromo-2,2′-bipyrrole
**(16)**	bromophenol-bromopyrrole pentabromopseudilin
**(17)**	2,3,5,7-tetrabromobenzofuro[3,2-*b*]pyrrole
**(18)**	2,3,4,5-tetrabromopyrrole
**(19)**	6-OH-BDE-47 or 6-hydroxy-2,2′,4,4′-tetrabromodiphenyl ether
**(20)**	Deca-BDE/BDE-209 or 2,2′,3,3′,4,4′,5,5′,6,6′-decabrominated diphenyl ether
**(21)**	BDE-47 or 2,2′,4,4′-tetrabrominated diphenyl ether
**(22)**	2-MeO-6-OH-BDE-68
**(23)**	2,6′-OH-BDE-68
**(24)**	Spongiadoxin C
**(25)**	Methylated Spongiadoxin C
**(26)**	Thyroxine (T_4)
**(27)**	3-OH-BDE-47
**(28)**	3′-OH-BDE-154 or 2,2′,4,4′,5,6′-hexabromodiphenyl ether
**(29)**	BDE-99 or 2,2′,4,4′,5-pentabromodiphenyl ether
**(30)**	BDE-153 or 2,2′,4,4′,5,5′-hexabromodiphenyl ether
**(31)**	2-(3′,5′-dibromo-2′-hydroxyphenoxy)-3,4,5,6-tetrabromophenol
**(32)**	2-(3′,5′-dibromo-2′-hydroxyphenoxy)-3,5,6-tribromophenol
**(33)**	2,4-dibromo-6-(2,4-dibromophenoxy)phenol
**(34)**	3,5-dibromo-2-(4′,5′,6′-tribromo-2′-hydroxyphenoxyphenol or<br>2-(4′,6′-dibromo-2′-hydroxyphenoxy)-3,4,5-tribromophenol
**(35)**	2,4-dibromo-6-(4-bromo-2-hydroxyphenoxy)phenol
**(36)**	3,5-dibromo-2-(2′,4′-dibromophenoxy)phenol or<br>2-(2′,4′-dibromophenoxy)-3,5-dibromophenol
**(37)**	4,6-dibromo-2-(2′,4′-dibromophenoxy)phenol) or<br>2-(2′,4′-dibromophenoxy)-4,6-dibromophenol or P01F03
**(38)**	3,5,6-tribromo-2-(2′-bromophenoxy)phenol
**(39)**	2-(2′,4′-dibromophenoxy)-3,4,5-tribromophenol or<br>3,4,5-tribromo-2-(2′,4′-dibromophenoxy)phenol
**(40)**	1-methoxy-2′-hydroxy-4,4′,5,6′-tetrabrominated diphenyl ether or<br>3,5-dibromo-2-(3,5-dibromo-2-methoxyphenoxy)phenol
**(41)**	4,6-dibromo-2-(4′,5′,6′-tribromo-2′-hydroxyphenoxy)phenol-1-methyl ether or<br>2′-methoxy-1-hydroxy-3,3′,4,5,5′-pentabrominated diphenyl ether
**(42)**	5-OH-BDE-47

**(43)**	PCB 47 or 2,2′,4,4′-tetrachlorbiphenyl
**(44)**	PCB 52 or 2,2′,5,5′-tetrachlorbiphenyl

Supplemental Figure S1. List of all compounds mentioned in text and figures with their corresponding Arabic numeral. The IUPAC name(s) of every compound, which is mentioned in the text and in this figure can be identified with its corresponding Arabic numeral in Supplemental Table S1.

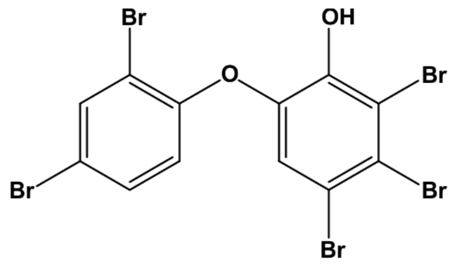

(1)

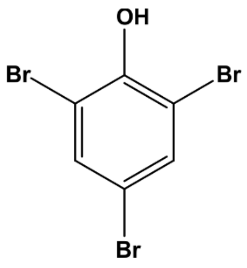

(7)

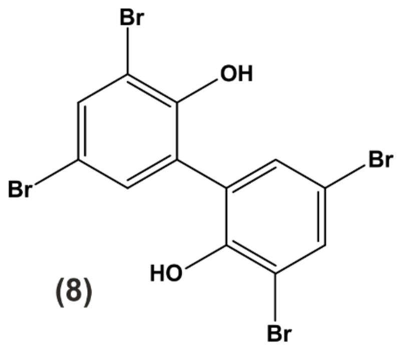

(8)

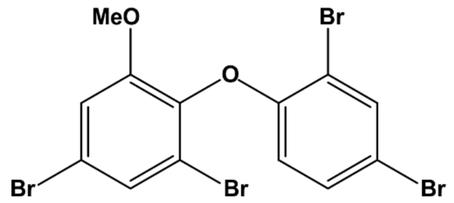

(2)

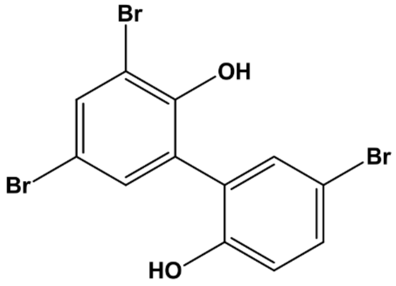

(9)

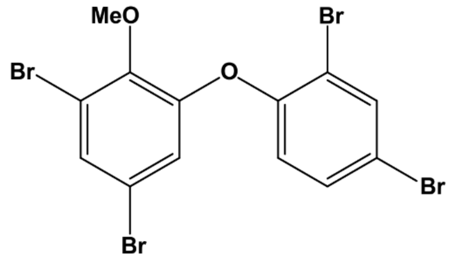

(3)

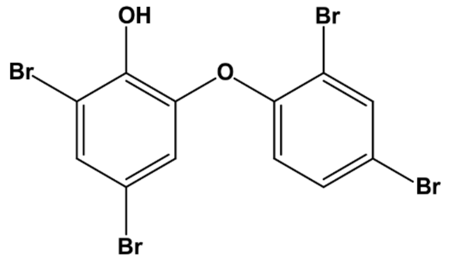

(10)

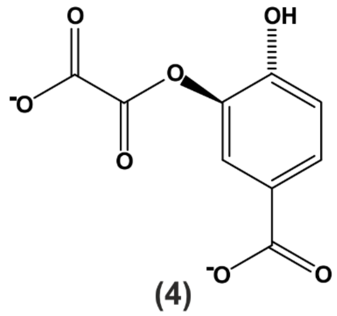

(4)

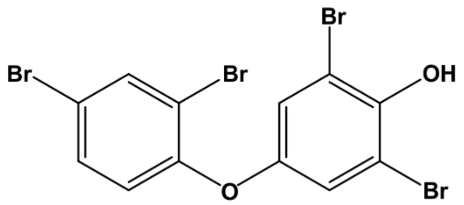

(11)

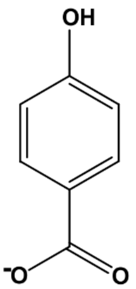

(5)

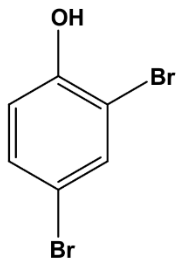

(6)

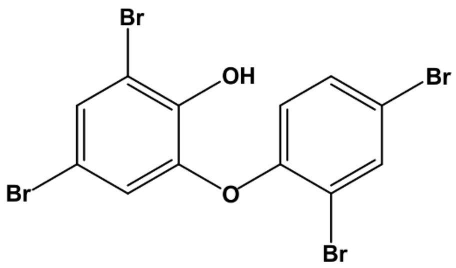

(12)

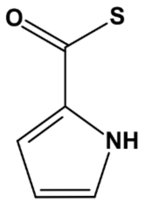

(13)

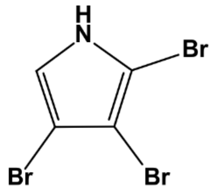

(14)

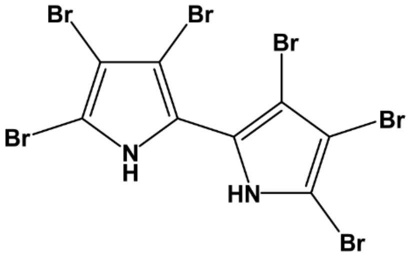

(15)

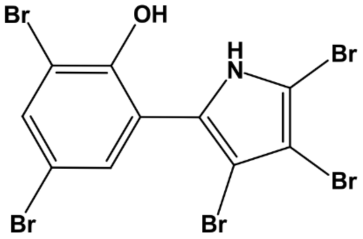

(16)

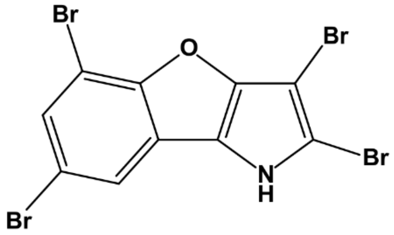

(17)

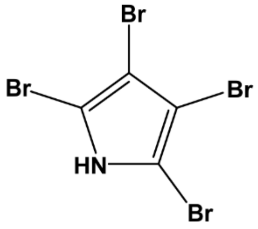

(18)

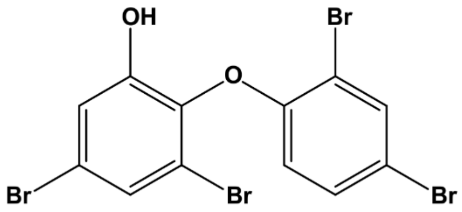

(19)

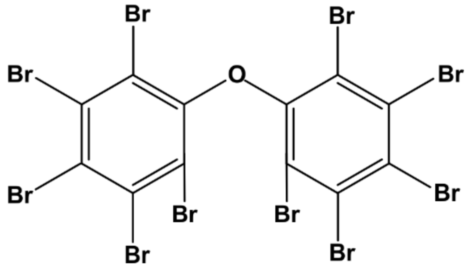

(20)

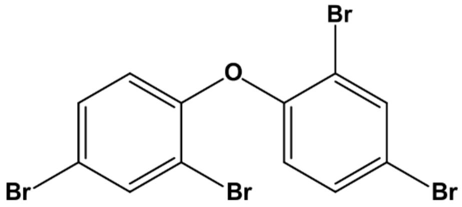

(21)

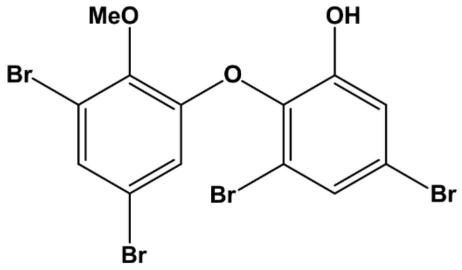

(22)

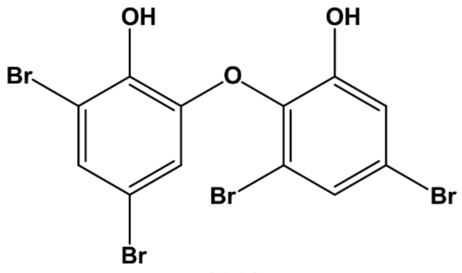

(23)

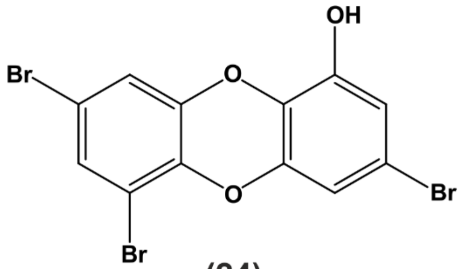

(24)

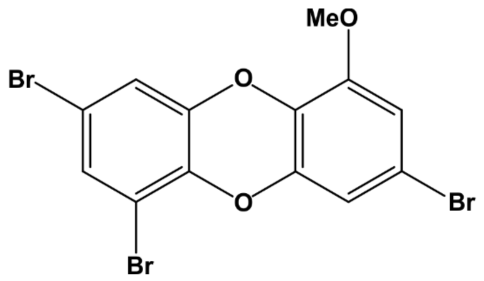

(25)

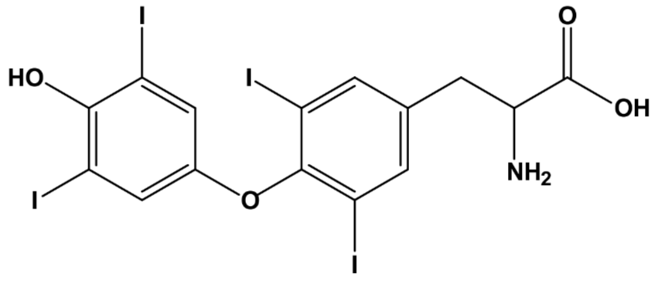

(26)

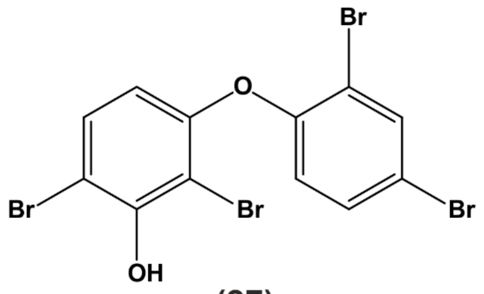

(27)

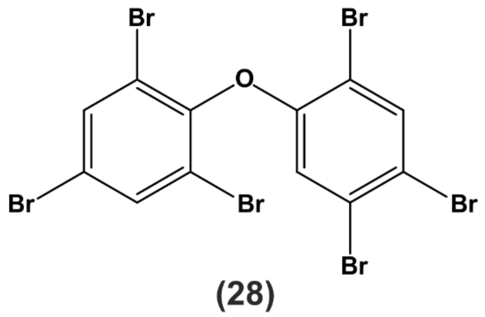

(28)

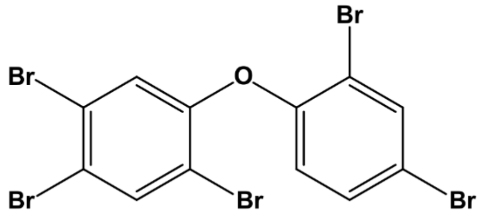

(29)

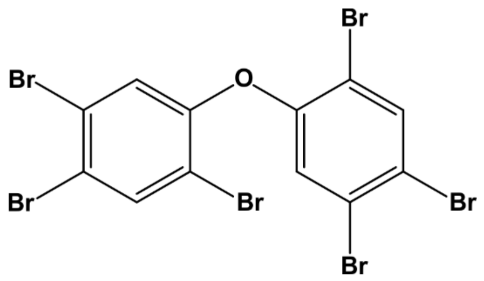

(30)

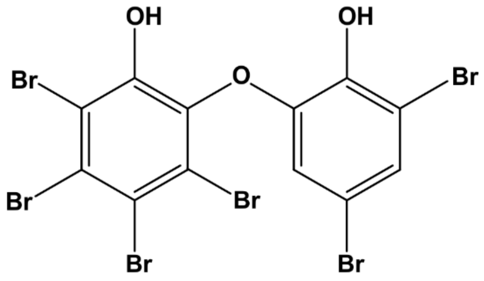

(31)

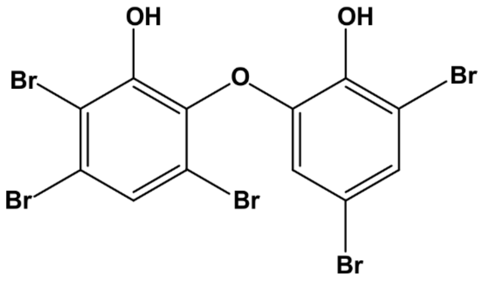

(32)

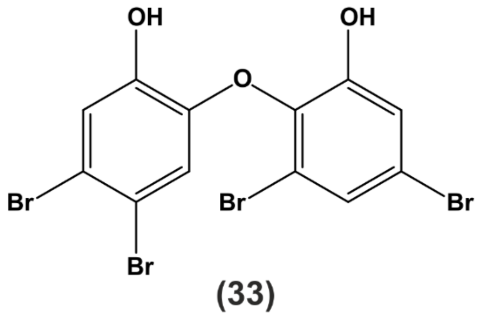

(33)

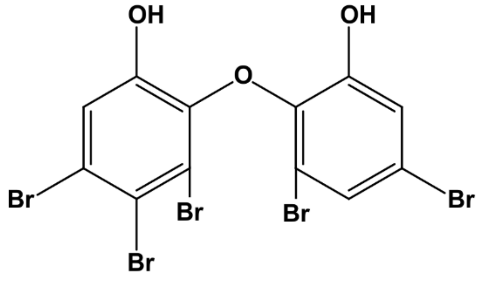

(34)

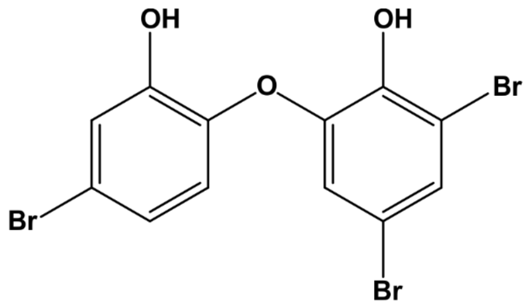

(35)

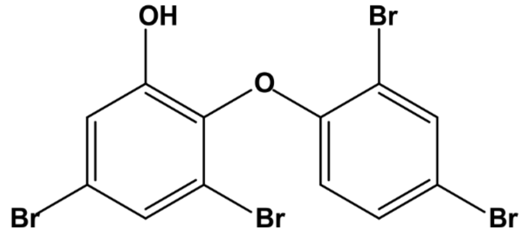

(36)

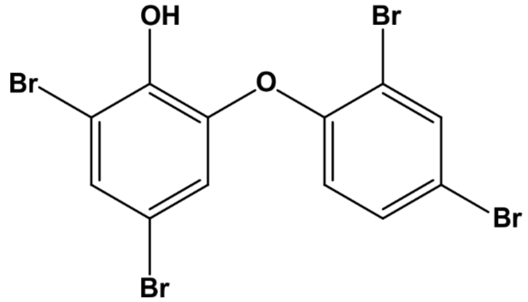

(37)

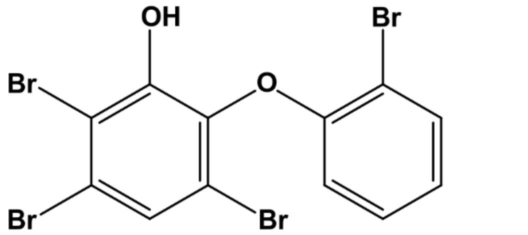

(38)

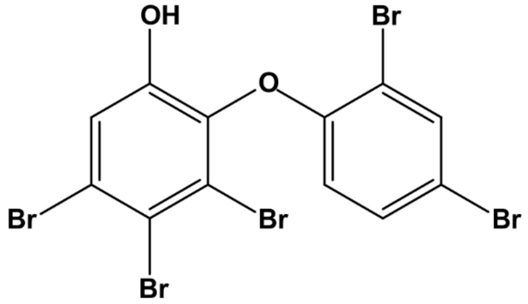

(39)

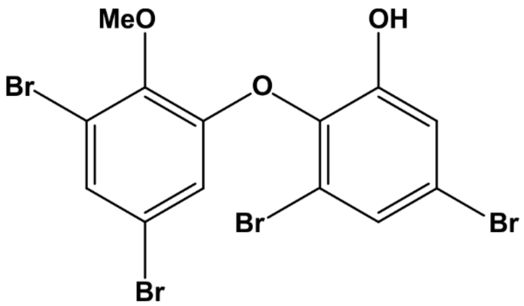

(40)

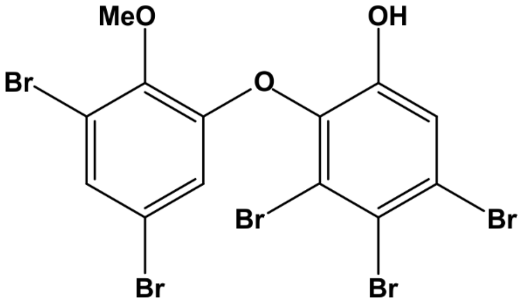

(41)

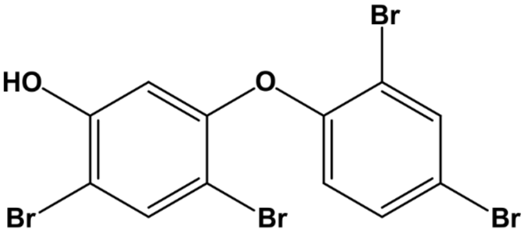

(42)

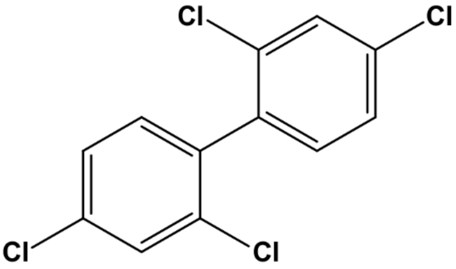

(43)

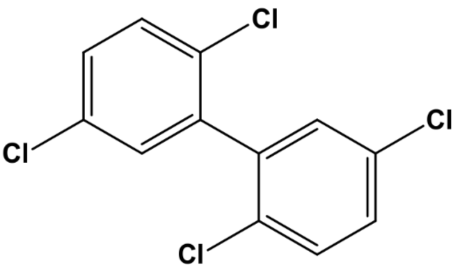

(44)
